# Redox Regulator *GLRX* Is Associated With Tumor Immunity in Glioma

**DOI:** 10.3389/fimmu.2020.580934

**Published:** 2020-11-30

**Authors:** Yuanhao Chang, Guanzhang Li, You Zhai, Lijie Huang, Yuemei Feng, Di Wang, Wei Zhang, Huimin Hu

**Affiliations:** ^1^ Department of Molecular Neuropathology, Beijing Neurosurgical Institute, Capital Medical University, Beijing, China; ^2^ Department of Neurosurgery, Beijing Tiantan Hospital, Capital Medical University, Beijing, China; ^3^ China National Clinical Research Center for Neurological Diseases, Beijing, China; ^4^ Chinese Glioma Genome Atlas Network (CGGA) and Asian Glioma Genome Atlas Network (AGGA), Beijing, China

**Keywords:** glioma, *GLRX*, macrophage, prognosis, tumor immunity

## Abstract

Glutaredoxin is central to cellular redox chemistry and regulates redox homeostasis and malignant progression of many cancers. In glioma, the role of its coding gene (*GLRX*) remains unclear. We aimed to elucidate the role of glutaredoxin at the transcriptome level and its clinical prognostic value in glioma. In total, we evaluated 1,717 glioma samples with transcriptome data and corresponding clinical data as well as single-cell sequencing data from 6 glioma patients from publicly available databases. Gene set variation analysis and gene ontology analysis were performed to reveal the biological function of *GLRX*. The immune cell enrichment score was calculated by GSVA analysis. Single-cell sequencing data was visualized by *t*-distributed stochastic neighbor embedding analysis. The prognostic value of *GLRX* in glioma was verified by the Kaplan-Meier curve and multivariate COX analysis. *GLRX* was found to be highly enriched in gliomas of higher grades with wild-type *IDH*, without 1p/19q co-deletion, and with a methylated *MGMT* promoter. Moreover, *GLRX* could be a potential marker for the mesenchymal molecular subtype of gliomas. The expression of *GLRX* was closely related to the tumor immune process, immune checkpoints, and inflammatory factors with *GLRX* being specifically expressed in M0 macrophages. *GLRX* is also shown to be an independent prognostic factor in glioma. Altogether, our study outcomes show that *GLRX* is highly enriched in malignant gliomas and is closely related to the tumor immune microenvironment. Therefore, *GLRX*-targeted cell redox regulatory therapy may enhance the efficacy of glioma immunotherapy.

## Introduction

Glioma is the most common malignant tumor affecting the central nervous system, and it is mainly characterized by a high recurrence rate and short survival time (1). To date, the most effective treatment for glioma is surgical resection to maximum safety extent (2), which can be followed by additional individualized treatments such as radiotherapy and chemotherapy. Even with aggressive treatment, the prognosis for glioma patients remains very poor. Therefore, finding novel therapeutic targets and molecular targeted drugs may pave the way for an improved prognosis for these patients.

Glutaredoxin (Grx), also known as thiol transferase, is ubiquitously expressed in bacteria, viruses, and mammals. It has a relative molecular weight of approximately 12 kDa and comprises 106–107 amino acids (3). Grx is an important component of the thiol-disulfide bond oxidoreductase family and catalyzes the redox reaction between glutathione (GSH) and protein disulfide bonds that are necessary for optimal protein activity (4). Several studies have reported that Grx performs a variety of biological functions in cancer related to relieving oxidative stress, transcription regulation, and control of DNA synthesis by modulating the activity of ribonucleotide reductase (3, 5, 6). However, there are few reports on the role of Grx in glioma.

The GSH system is an essential regulator of redox balance in the brain (7), and Grx acts as a central “antioxidant” in neurons to protect them from oxidative stress injury. Previous studies have reported that Grx is also involved in glioma and metastasis development as well as in drug resistance (6, 8). Therefore, understanding the role of Grx in the context of glioma is pivotal for the development of novel therapeutic approaches targeting malignant gliomas.

We investigated the expression and function of the Grx coding gene (*GLRX*) at the transcriptome level using publicly available data sets from the Chinese Glioma Genome Atlas (CGGA) and The Cancer Genome Atlas (TCGA), which included RNA sequencing (RNA-seq) data and corresponding clinical details about the cancer patients. We found that *GLRX* is associated with high tumor grade and malignant phenotypes. Moreover, gene ontology analysis and gene set variation analysis revealed, for the first time, that *GLRX* can function as a mediator of the immune response. Further CIBERSORT analysis revealed that a higher expression level of GLRX is correlated with enrichment of macrophages in glioma tissue. Single-cell analysis, immunohistochemical (IHC) staining, and immunofluorescent staining (IF) validated that GLRX may be specifically expressed in M0 macrophages. Last, we found that *GLRX* is an independent prognostic factor in glioma. Altogether, these findings suggest that *GLRX* is highly enriched in malignant gliomas and is closely related to the tumor immune microenvironment. Therefore, *GLRX*-targeted cell redox regulatory therapy may enhance response to immunotherapy in patients with glioma.

## Materials and Methods

### Data Collection

This study was approved by the Capital Medical University Institutional Review Board. We collected transcriptome sequencing data generated by the Illumina HiSeq platform that was publicly available from the CGGA and CGGA (2019) databases (https://www.cgga.org.cn) for 325 and 693 samples, respectively. We evaluated the status of isocitrate dehydrogenase (IDH) mutation, 1p/19q, and MGMT promoter methylation as described in previous studies (9–11). Overall patient survival was estimated from the date of diagnosis to the reported date of death or last follow-up. RNA-seq data were obtained from TCGA (https://tcgadata.nci.nih.gov), and single-cell sequencing data were retrieved from the GSE89567 data set of the Gene Expression Omnibus database. All clinical and molecular information on the samples evaluated in the present study is presented in **Table 1**. We used the online software GEPIA (http://gepia.cancer-pku.cn) (12) to evaluate expression differences between glioblastoma multiform (GBM) and normal brain tissues.

**Table 1:** Sample information.

Characteristics (CGGA)	No. of Patients (n=325)
*Age*	
<45	191
≥45	134
*Gender*	
Male	203
Female	122
*WHO Grade*	
Grade II	103
Grade III	79
Grade IV	139
NA	4
*TCGA Subtypes*	
Proneural	102
Neural	81
Classical	74
Mesenchymal	68
*Radiotherapy+TMZ Chemotherapy*	
Yes	154
No	24
*Radiotherapy*	
Yes	258
No	51
NA	16
*TMZ Chemotherapy*	
Yes	178
No	124
NA	23
*IDH1/2 mutation*	
Mutation	175
Wild type	149
NA	1
*1p/19q codeletion*	
Co-deletion	67
Non-co-deletion	250
NA	8
*MGMT methylation*	
Methylation	130
Unmethylation	112
NA	64
**Characteristics [CGGA(2019)]**	**No. of Patients (n=693)**
*Age*	
<45	382
≥45	310
NA	1
*Gender*	
Male	398
Female	295
*WHO Grade*	
Grade II	188
Grade III	255
Grade IV	249
NA	1
*TCGA Subtypes*	
Proneural	296
Neural	167
Classical	83
Mesenchymal	147
*Radiotherapy+TMZ Chemotherapy*	
Yes	413
No	67
*Radiotherapy*	
Yes	509
No	113
NA	71
*TMZ Chemotherapy*	
Yes	457
No	151
NA	85
*IDH1/2 mutation*	
Mutation	356
Wild type	286
NA	51
*1p/19q co-deletion*	
Co-deletion	145
Non-co-deletion	478
NA	70
*MGMT methylation*	
Methylation	127
Unmethylation	73
NA	492
**Characteristics (TCGA)**	**No. of Patients (n=699)**
*Age*	
<45	296
≥45	340
NA	63
*Gender*	
Male	368
Female	268
NA	63
*WHO Grade*	
Grade II	223
Grade III	245
Grade IV	168
NA	63
*TCGA Subtypes*	
Proneural	250
Neural	115
Classical	92
Mesenchymal	105
NA	137
*IDH1/2 mutation*	
Mutation	443
Wild type	246
NA	10
*1p/19q co-deletion*	
Co-deletion	172
Non-co-deletion	520
NA	7
*MGMT methylation*	
Methylation	492
Unmethylation	168
NA	39

The number of glioma patients engaged in our study is listed. All patients were stratified with age, clinicopathological characteristics, and treatment options, respectively.

### Gene Ontology (GO) and Kyoto Encyclopedia of Genes and Genomes (KEGG) Analyses

The biological functions and signaling pathways related to *GLRX* were explored by GO and KEGG analyses using the DAVID bioinformatics resource (version 6.7) (13). After Spearman correlation analysis, GO results on the most correlated genes were visualized by heat map.

### Gene Set Variation Analysis (GSVA)

GSVA was performed with the GSVA package (from R Project 3.5.1) of R software with default parameters. The list of GO terms was obtained from the Gene Set Enrichment Analysis database (https://www.gsea-msigdb.org/gsea/msigdb/index.jsp). Relationships between genes and biological functions were determined using Pearson correlation analysis.

### Immune Function Analysis

The relationship between *GLRX* expression and immune function was evaluated by Pearson correlation analysis. Immune function scores (14) were calculated by GSVA analysis, and the immune function gene set was downloaded from AmiGO 2 (http://amigo.geneontology.org/amigo/landing). The classification of immune functions was done according to the guidelines of AmiGO 2.

### CIBERSORT

RNA-seq data were evaluated using the CIBERSORT software (https://cibersort.stanford.edu). The signature gene profile of 22 immune cell types was used in CIBERSORT to estimate the proportion of tumor-infiltrating immune cell types (15).

### T-Distributed Stochastic Neighbor Embedding (T-SNE) Analysis

T-SNE analysis was performed with the Rtsne package from R Project (version 3.5.1); perplexity was set to 20. Identification of cell types was based on the specific cell markers obtained from the CellMarker database (http://biocc.hrbmu.edu.cn/CellMarker/).

### Prognostic Analysis

Patient survival distribution and significance was evaluated by Kaplan-Meier survival curve and log-rank test. Kaplan-Meier analysis was performed using R software (version 3.5.1, http://www.r-project.org). The prognostic value of *GLRX* was estimated by univariate and multivariate Cox proportional hazard model analysis using SPSS statistical software (version 25.0; IBM, Armonk, NY, USA). Patients with missing information were excluded from the analysis.

### IHC Staining

Paraffin-embedded samples were obtained from the CGGA sample bank. First, 5-µm sections were cut for IHC staining. Samples were deparaffinized in an oven at 65°C for 1 h. Then the samples were rehydrated in decreasing concentrations of alcohol. IHC analysis with GLRX1 (Abcam, ab45953,1:1000) and CD11b antibodies (Proteintech, 66519-1-lg, 1:1000) was conducted according to standard procedures. Photos were taken with an optical microscope.

### Cell Culture and Reagents

THP-1 cells (purchased from National Infrastructure of Cell Line Resource, http://www.cellresource.cn/) were maintained in RPMI1640 media supplemented with L-glutamine, 1% penicillin and streptomycin, β-mercaptoethanol (Gibco, 2169148, 0.055 mM) and 10% fetal bovine serum (FBS, Gibco) at 37°C under a humidified, 5% CO_2_ atmosphere (16). THP-1 cells were differentiated to M0 macrophages by treatment with 25 nM phorbol 12-myristate 13-acetate (MCE, HY-18739) for 48 h, washed and incubated with normal RPMI1640 media for 24 h, and then incubated with recombinant human GM-CSF (50 ng/ml, Peprotech, 300-03) for 96 h. For M2 polarization, 50% of complete RPMI1640 medium was added, and it was incubated for 48 h. Then the M2 macrophage was obtained by removing the culture medium and culturing cells for an additional 48 h in M2 medium with recombinant human M-CSF (100 ng/ml, Peprotech, 300-25) (17).

### IF Staining

Macrophages were washed with PBS three times. Four percent fixative solution (Solarbio, P1110) was added to the Petri dish for 10 min. Then, the solution was removed and cells washed three times. Next, 0.5% Triton X-100 (Solarbio, T8200) was added to the dish for 10 min. The solution was removed and cells washed three times. Five percent BSA (Solarbio, A8010) was added to the dish, and it was incubated for 1 h at room temperature. Then, primary antibodies were added to the M0 (GLRX1: 1:500, Abcam, ab45953; CD11b: 1:100, Proteintech, 66519-1-lg) and M2 macrophages (GLRX1: 1:500, Abcam, ab45953; CD163: 1:100, Abcam, ab156769) (18), respectively, and they were incubated overnight at 4°C. The solution was removed and cells washed three times. Secondary antibodies (DyLight 488 goat antirabbit polyclonal antibody, Abcam, ab96899, 1:200; DyLight 594 goat antimouse polyclonal antibody, Abcam, ab96881, 1:200) were used for 1 h at room temperature. The solution was removed and cells washed three times. Prolong™ Diamond Antifade Mountant with DAPI (Invitrogen, P36962) was added to the dish, and photos were taken with confocal microscopy.

### Other Immune Biological Analysis

Pearson’s correlation analysis was used to evaluate the relationship between *GLRX* and immune checkpoints. Inflammation-related metagenes were described as before (19).

### Statistical Analysis

A multiple group comparison was performed using Tukey’s test. Other statistical computations and figure drawing were performed with several R packages, including ggplot2, pheatmap, pROC, and corrgram. All statistical tests were two-sided, and a p-value < 0.05 was considered statistically significant in all analyses.

## Results

### Association of *GLRX* Expression With Clinical and Molecular Pathological Characteristics in Glioma

**Figure 1:**
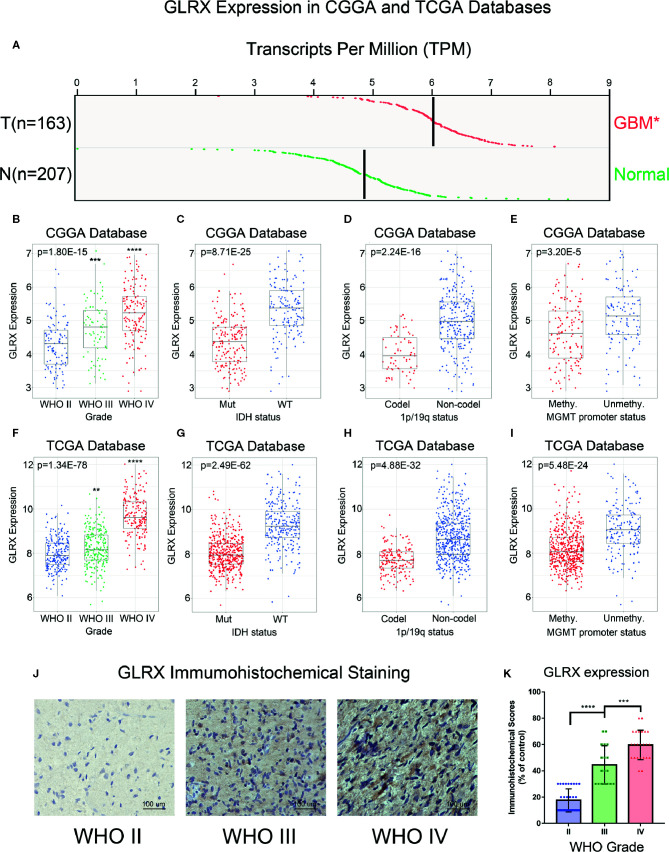
*GLRX* is correlated with relative malignant molecular pathological characteristics of gliomas. **(A)** *GLRX* showed a significantly higher expression in GBM (WHO grade IV) compared with normal brain tissue in online analysis of GEPIA (T: GBM tumor; N: normal brain tissue). **(B, F)** *GLRX* was significantly increased in GBM (WHO grade IV) in the CGGA and TCGA databases. **(C, G)** *GLRX* was significantly increased in IDH wild-type gliomas in the CGGA and TCGA databases (Mut: IDH mutation; WT: IDH wildtype). **(D, H)** *GLRX* was significantly increased in 1p/19q non-co-deletion gliomas in the CGGA and TCGA databases (Codel: 1p/19q co-deletion; Non-codel: 1p/19q non-co-deletion). **(E, I)** *GLRX* was significantly increased in the MGMT unmethylated group in the CGGA and TCGA databases. **(J)** The representative photos of IHC staining of GLRX in different glioma grades. Scale bar is 100 um. **(K)** The immunohistochemical scores of GLRX were measured in different grades. Respectively, 32, 18, and 24 patients were from grade II, grade III and grade IV. *, **, ***, and **** indicate p < 0.05, p < 0.01, p < 0.001, and p < 0.0001, respectively.

To investigate the role of *GLRX* in gliomas, we compared the expression levels of *GLRX* between normal brain tissue and GBM (grade IV, according to the World Health Organization [WHO]). The analysis revealed that *GLRX* expression was significantly enriched in GBM samples (p < 0.05, **Figure 1A**). Due to the histopathological heterogeneity of gliomas, RNA-seq data of glioma samples from three independent databases were analyzed according to WHO guidelines, and the analysis included *IDH* mutation status, 1p/19q co-deletion status, and MGMT promoter status. Among samples from the CGGA database, *GLRX* expression was higher in GBM (grade IV) compared with glioma (grades II and III) (**Figure 1B**). This result was further validated in the RNA-seq data from TCGA and CGGA (2019) databases (**Figure 1F** and **Supplementary Figure S1A**). In addition, IHC staining was conducted to explore the expression of *GLRX* in glioma tissues. Consistent with the RNA-seq data, we found that *GLRX* was enriched in GBM tissues (**Figures 1J, K**). The *IDH* mutation status, 1p/19q co-deletion status, and MGMT promoter status play important roles in the prognosis and chemotherapy outcomes of glioma patients and vary significantly among glioma patients (20). Therefore, we explored the correlation between *GLRX* expression and these three molecular pathologic statuses. We found that *GLRX* expression was highly enriched in *IDH* wild-type glioma patients compared with those harboring *IDH* mutations (**Figures 1C, G** and **Supplementary Figure S1B**). Moreover, patients with 1p/19q non-co-deletion had a higher expression of *GLRX* in all three databases (**Figures 1D, H** and **Supplementary Figure S1C**). Regarding the MGMT promoter status in the CGGA database, we found that gliomas with a methylated MGMT promoter had lower *GLRX* expression compared to those in the unmethylated group (**Figure 1E**). A similar trend was observed in the two other databases (**Figure 1I** and **Supplementary Figure S1D**). These findings indicate that *GLRX* expression is enriched in GBM and is tightly correlated with the malignant phenotype of glioma.

### *GLRX* Is a Potential Marker for Mesenchymal Molecular Subtype Glioma

**Figure 2:**
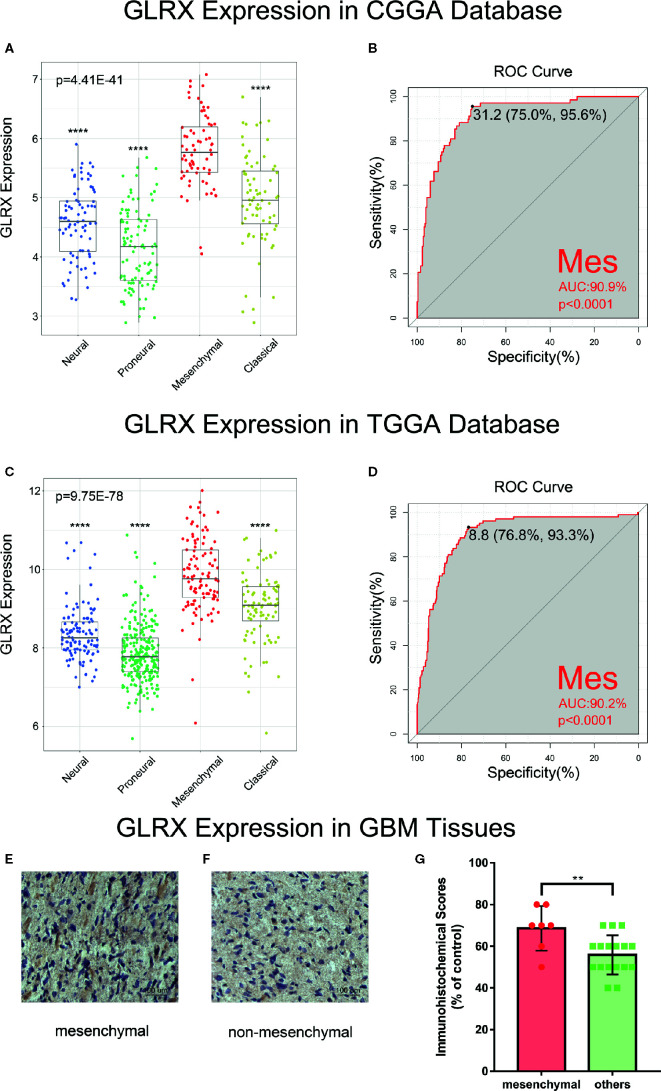
*GLRX* is a potential marker for malignant subtypes of gliomas. **(A, C)** *GLRX* was highly expressed in the mesenchymal subtype in the CGGA and TCGA databases. **(B, D)** ROC curve analysis showed that *GLRX* was highly sensitive and specific to predict the mesenchymal subtype in the CGGA and TCGA databases. **(E, F)** The representative photos of IHC staining of GLRX in different TCGA molecular subtypes. Scale bar is 100 um. **(G)** The immunohistochemical scores of GLRX were measured in different TCGA molecular subtypes. Respectively, 7 and 17 patients were from GBM. Differences between groups were tested by Tukey’s multiple comparisons test. ** and **** indicate p<0.01 and p < 0.0001, respectively.

**Figure 3:**
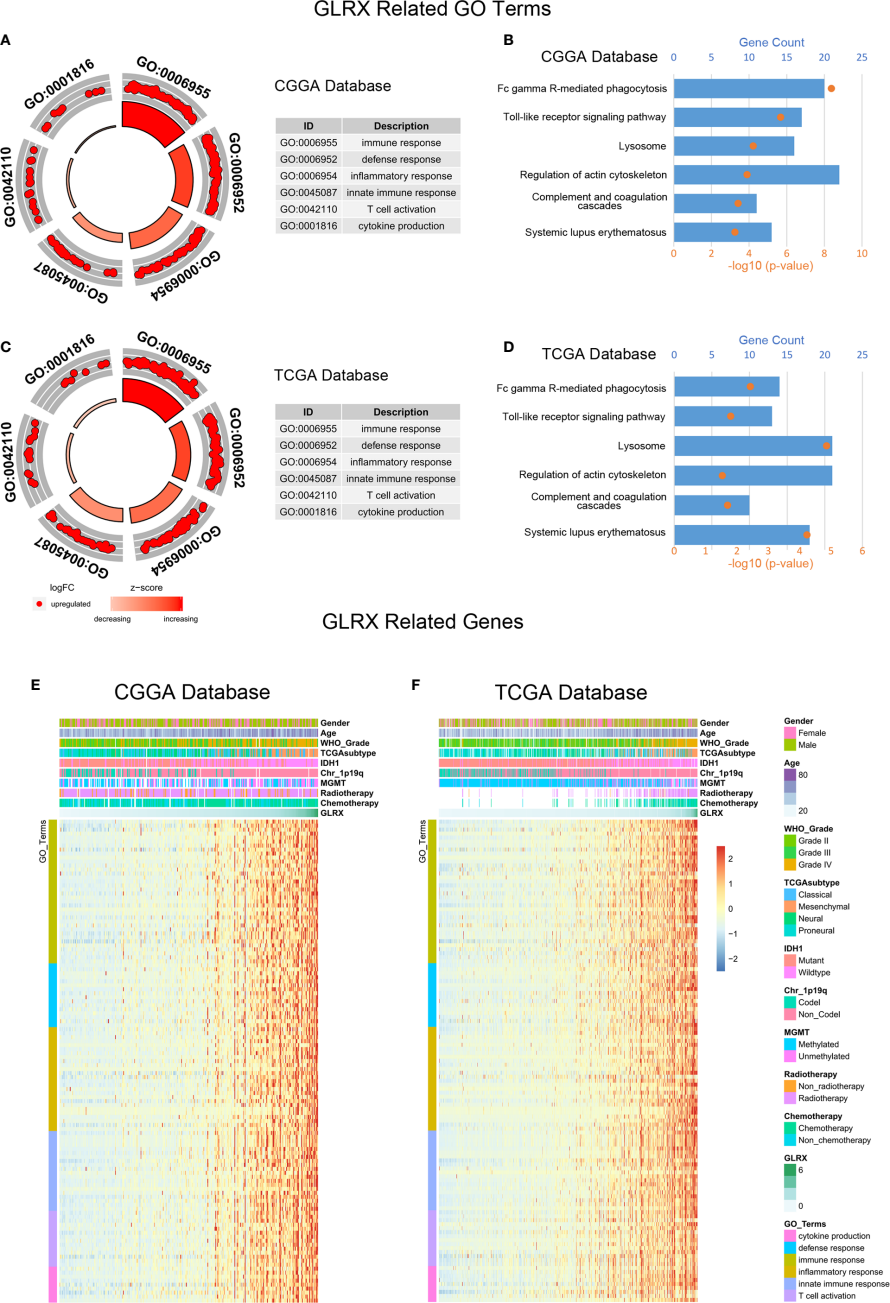
*GLRX* is strongly associated with immune processes in gliomas. **(A, C)** GO analysis showed that *GLRX* was mostly associated with immune, defense, and inflammatory responses in both the CGGA and TCGA databases. **(B, D)** KEGG pathway analysis showed that *GLRX* was mostly involved in the immune response–related pathway in the CGGA and TCGA databases. **(E, F)** Most immune process–related genes were significantly positively correlated with *GLRX* expression in the CGGA and TCGA databases.

Next, we investigated the molecular expression pattern of *GLRX* in different molecular subtypes defined by TCGA network (21). *GLRX* was significantly upregulated in the mesenchymal subtype of glioma compared with the other three subtypes in the CGGA (**Figure 2A**), TCGA (**Figure 2C**), and CGGA (2019) databases (**Supplementary Figure S2A**). The IHC staining of *GLRX* in GBM tissues also verified this finding (**Figures 3E–G**). To further validate this finding, we evaluated the receiver operating characteristic (ROC) curve for *GLRX* expression and mesenchymal subtype for gliomas of all grades. Surprisingly, the area under the curve (AUC) of *GLRX* expression was up to 90.9%, 90.2%, and 78.0% for the CGGA, TCGA, and CGGA (2019) data sets, respectively (**Figures 2B, D** and **Supplementary Figure S2B**). These results suggest that GLRX is highly expressed in mesenchymal subtype glioma and may play an oncogenic role in glioma progression. BMI1 and CD44 were reported to differentiate the mesenchymal molecular subtype from other gliomas (22, 23). Thus, we took these two well-studied biomarker genes as positive controls to performed ROC curve analysis (**Supplementary Figure S2C–H**). Through comparing the AUC of these three genes, we inferred that *GLRX* may serve as a biomarker for mesenchymal subtype gliomas.

### *GLRX* Is Strongly Associated With Immune Functions in Glioma

We performed GO analysis to identify the *GLRX*-related biological functions in gliomas. At first, we screened genes that were strongly correlated with *GLRX* (Pearson R > 0.55 and p < 0.0001) in all three databases. The analysis revealed a total of 479 genes in CGGA, 877 genes in TCGA, and 537 genes in CGGA (2019) that were significantly correlated with *GLRX* expression. The genes positively correlated with *GLRX* expression were mostly involved in immune response, defense response, and inflammatory response in all databases (**Figures 3A, C** and **Supplementary Figure S3A**). Additionally, we performed KEGG pathway analysis to further explore the signaling pathways associated with the abovementioned genes. As expected, the KEGG analysis identified these genes to be associated with immune response pathways, including FcγR-mediated phagocytosis, the toll-like receptor signaling pathway, and complementary and coagulation cascades in the three databases (**Figures 3B, D** and **Supplementary Figure S3B**). The heat map representation of the genes (shown in **Table 2**) within each biological process exhibits a clear positive correlation with *GLRX* expression and the landscape of corresponding clinical patient features (**Figures 3E, F** and **Supplementary Figure S3C**). These findings suggest that *GLRX* takes part in the immune response process and may be a marker for predicting immune-related biological processes in gliomas.

**Table 2:** Representative genes of each biological function.

Gene	GO_Terms
NFKB2	immune response
B2M	immune response
IL4R	immune response
LILRA6	immune response
FCGR3A	immune response
LAIR1	immune response
DBNL	immune response
NCF4	immune response
STXBP2	immune response
TNFRSF14	immune response
CTSS	immune response
PDCD1LG2	immune response
BCAP31	immune response
LILRB1	immune response
LAT2	immune response
CTSC	immune response
GBP2	immune response
GALNT2	immune response
SBNO2	immune response
IFITM3	immune response
GPSM3	immune response
GPR65	immune response
FCGRT	immune response
FTH1	immune response
SQSTM1	immune response
FCER1G	immune response
MR1	immune response
ARHGDIB	immune response
PSMB8	immune response
TNFSF8	immune response
PSMB9	immune response
IKBKE	immune response
FCGR2B	immune response
CD300A	immune response
CD274	immune response
RNF19B	immune response
TNIP1	defense response
C5AR1	defense response
CLIC1	defense response
SP140	defense response
MNDA	defense response
CLEC5A	defense response
TYROBP	defense response
TCIRG1	defense response
HCK	defense response
MAP2K3	defense response
CD300C	defense response
APOL2	defense response
CYBB	defense response
STAB1	defense response
ALOX5	defense response
CD14	defense response
CCL2	inflammatory response
NMI	inflammatory response
ADORA3	inflammatory response
S100A8	inflammatory response
AIF1	inflammatory response
CCR1	inflammatory response
S100A9	inflammatory response
ITGB2	inflammatory response
TNFRSF1B	inflammatory response
IL10RB	inflammatory response
HMOX1	inflammatory response
TICAM2	inflammatory response
SERPINA3	inflammatory response
C2	inflammatory response
SPP1	inflammatory response
B4GALT1	inflammatory response
NFKBIZ	inflammatory response
CEBPB	inflammatory response
LY96	inflammatory response
PDPN	inflammatory response
LYZ	inflammatory response
NFAM1	inflammatory response
IL6R	inflammatory response
CD40	inflammatory response
CD163	inflammatory response
CCR5	inflammatory response
KYNU	innate immune response
IL1R1	innate immune response
TLR1	innate immune response
NCF1C	innate immune response
TLR2	innate immune response
C1R	innate immune response
APOBEC3G	innate immune response
C1S	innate immune response
C1QC	innate immune response
GCH1	innate immune response
SP100	innate immune response
NCF2	innate immune response
NCF1	innate immune response
SERPING1	innate immune response
C1QA	innate immune response
CYBA	innate immune response
C1QB	innate immune response
CORO1A	innate immune response
C1RL	innate immune response
VSIG4	innate immune response
PTPRC	T cell activation
STAT5A	T cell activation
RELB	T cell activation
PTPN22	T cell activation
VAV1	T cell activation
ITGAM	T cell activation
DOCK2	T cell activation
CD86	T cell activation
IRF1	T cell activation
CLEC7A	T cell activation
FAS	T cell activation
LCP1	T cell activation
RAB27A	T cell activation
SYK	T cell activation
SLC11A1	cytokine production
NLRC4	cytokine production
MYD88	cytokine production
LYN	cytokine production
CD4	cytokine production
CD226	cytokine production
PRKCD	cytokine production
PTAFR	cytokine production
LCP2	cytokine production

The representative genes of each biological function are which obtained from GO analysis in heat map are listed.

### Special Immune Function of *GLRX*

**Figure 4:**
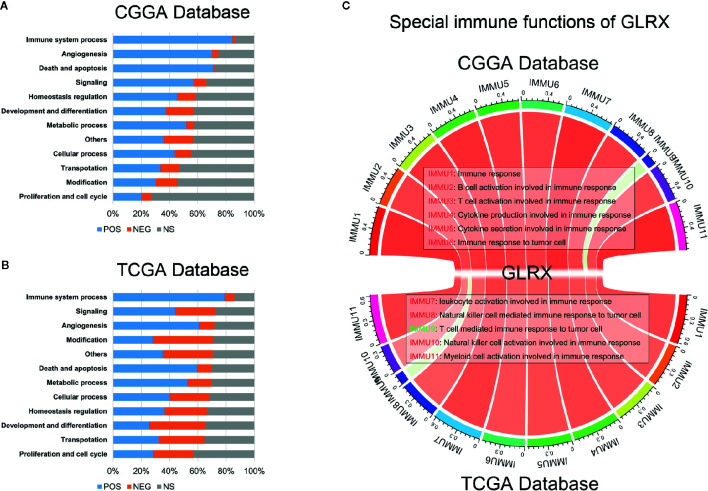
*GLRX* is closely related to the state of tumor immune functions. **(A, B)** *GLRX* had a positive correlation with 84.69% and 78.90% of the biological functions of immune system processes in the CGGA and TCGA databases, respectively. The scale values in the graph represent the proportions of significantly correlated biological functions in each biological function classification. **(C)** The correlation coefficient between *GLRX* and immune function scores in CGGA and TCGA databases. The red words represent a positive correlation. The green words represent a negative correlation.

Tumor-infiltrated immune cells, including T cells, NK cells, macrophages, and other cells, mount the immune response to kill or induce apoptosis of cancer cells (24). To further clarify the role of *GLRX* in the immune response in gliomas, we first assessed the correlation between *GLRX* and GO terms downloaded from the AmiGO2 web portal (http://amigo.geneontology.org/). We found 84.69%, 78.90%, and 87.07% biofunction of the immune system to be positively correlated with *GLRX* in the CGGA, TCGA, and CGGA (2019) data sets, respectively (**Figures 4A, B** and **Supplementary Figure S4A**). Overall, more immune-related GO terms were positively correlated with *GLRX* than any other kind of GO term. This further illustrates that *GLRX* has a strong correlation with the immune system. Last, to understand the role of *GLRX* in the immune system, we performed a correlation coefficient analysis on data from the three databases (**Figure 4C** and **Supplementary Figure S4B**). We observed that the majority of immune functions showed positive correlation with *GLRX*; only the term “T cell-mediated immune response to tumor cell (T cell response)” was found to be negatively correlated with *GLRX*.

### *GLRX* Is Associated With Inhibitory Immune Checkpoints and Inflammatory Responses

**Figure 5:**
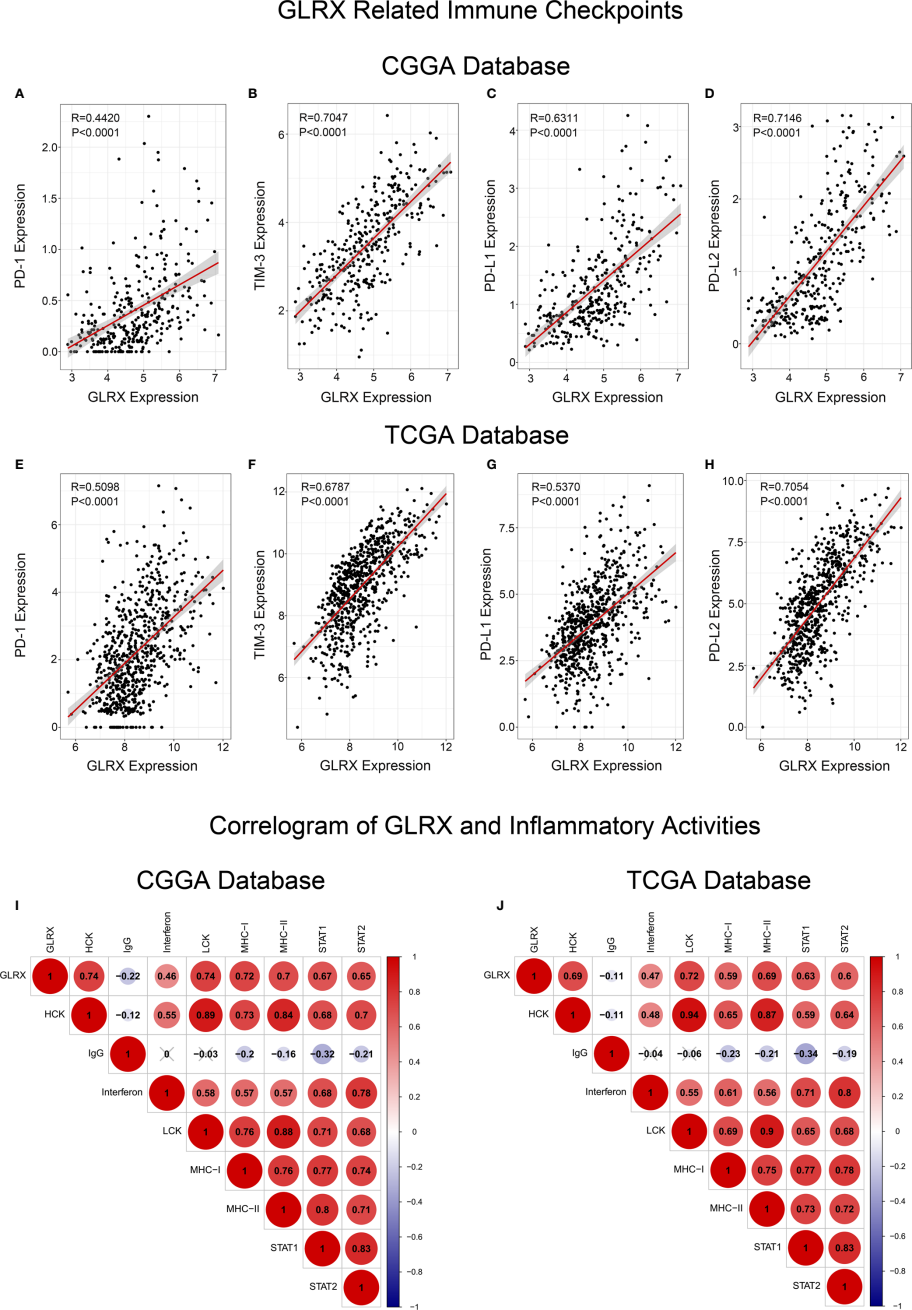
*GLRX* is associated with inhibitory immune checkpoints and inflammatory activities. **(A–H)** *GLRX* was synergistic with inhibitory immune checkpoints in tumor-induced immune responses. A strong correlation between *GLRX* and inhibitory immune checkpoint expression was found in both the CGGA and TCGA databases. **(I, J)** The correlation coefficient between *GLRX* and inflammatory activity function scores in gliomas. The red circle represents a positive correlation. The blue circle represents a negative correlation. The grey “×” represents no significant correlation. Similar results are found in both CGGA and TCGA databases.

As mentioned above, most immune functions had positive correlation with *GLRX* with the exception of T cell responses. In a previous study, we reported that glioma patients with a stronger immune response had a much poorer prognosis (25). This abnormal phenomenon suggests that depletion of immune components in gliomas triggered by immune checkpoints could contribute to a malignant tumor phenotype. To validate this hypothesis, we investigated the relationship between *GLRX* and known immune checkpoint genes, including PD-1, TIM-3, PD-L1, and PD-L2, in the CGGA, TCGA, and CGGA (2019) databases (**Figures 5A–H** and **Supplementary Figure S5A-D**). The results indicated that *GLRX* had a strong positive correlation with these inhibitory immune checkpoint molecules and that *GLRX* may influence their expression to support glioma cells escaping immunological surveillance. Additionally, we also analyzed the role of *GLRX* in the glioma inflammatory response in these databases as described previously (19) (**Figures 5I, J** and **Supplementary Figure S5E**). We found that *GLRX* was positively associated with HCK, interferons, LCK, MHC-I, MHC-II, STAT1, and STAT2 expression, and it was negatively associated with IgG expression. These results suggest that upregulation of *GLRX* is involved in the activation of signal transduction in T cells, macrophages, and antigen-presenting cells, but it is negatively associated with B lymphocytes related metagenes. All these findings collectively confirm that *GLRX* plays an important role in immune response in gliomas.

### *GLRX* Is Associated With M0 Macrophages

**Figure 6:**
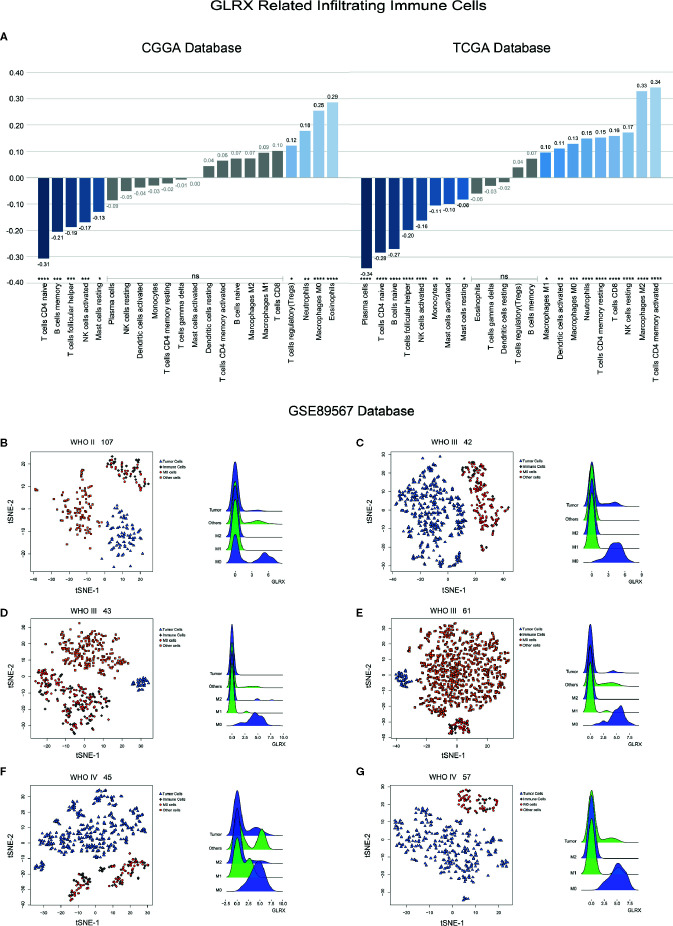
*GLRX* is expressed by immune cells. **(A)** The relationship between *GLRX* and infiltrated immune cells in both CGGA and TCGA databases. **(B–G)** The relationship between *GLRX* and macrophages in the GSE89567 database. The correlation between *GLRX* and infiltrated immune cells was analyzed by Pearson correlation analysis. ns, *, **, ***, and **** indicate no statistical difference, p < 0.05, p < 0.01, p < 0.001, and p < 0.0001, respectively. M0, M1, and M2 indicate M0, M1, and M2 macrophages, respectively.

**Figure 7:**
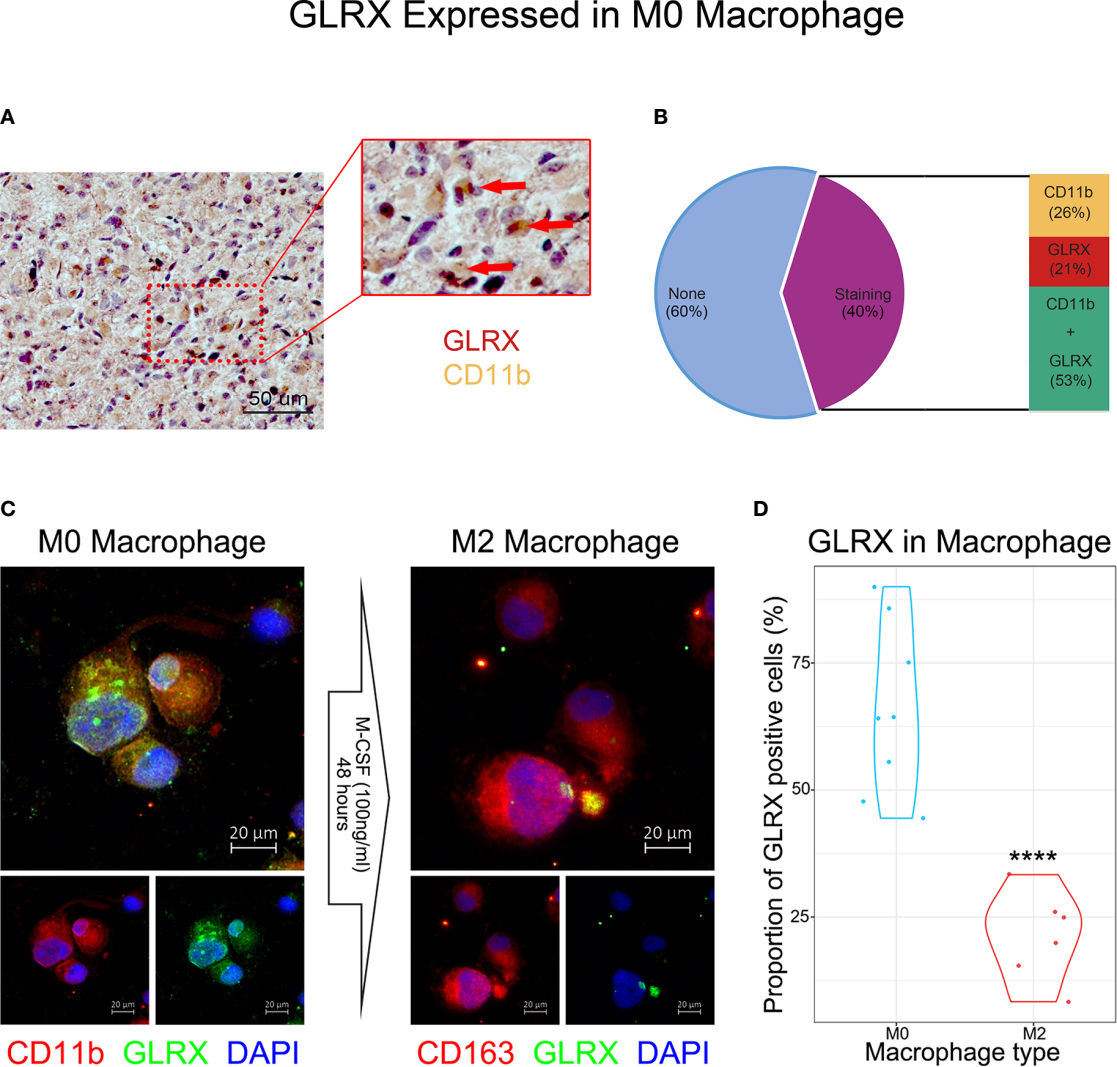
*GLRX* is specifically expressed by M0 macrophages. **(A)** The IHC co-localization staining of CD11b and GLRX. The red point represents GLRX, and the yellow area represents CD11b. Scale bar is 50 um **(B)** The proportion of stained cells. None represents cells without expression of CD11b and GLRX. Staining represents cells expressing CD11b and/or GLRX. CD11b represents cells expressing CD11b. GLRX represents cells expressing GLRX. CD11b + GLRX represents cells expressing CD11b and GLRX. **(C)** The left three photos are IF staining of M0 macrophages. Green fluorescence is GLRX, and red fluorescence is CD11b. Above is the merged photo of the below two. The right three photos are IF staining of M2 macrophages. Green fluorescence is GLRX, and red fluorescence is CD163. Above is the merged photo of the below two. Cell nuclei are stained with DAPI. Scale bar is 20 um. **(D)** The violin graph shows the proportion of GLRX-positive cells in M0 or M2 macrophage IF images. ****indicates p < 0.0001.

An activated immune response may promote the infiltration of immune cells into the tumor microenvironment and change its dynamics. To investigate whether *GLRX* was associated with infiltrated immune cells, we used CIBERSORT software to analyze the CGGA, TCGA, and CGGA (2019) databases (**Figure 6A** and **Supplementary Figure S6**). The analysis revealed that higher *GLRX* expression was positively correlated with enrichment of macrophages in glioma tissue. Moreover, single-cell sequencing data (**Figures 6B–G**) demonstrated that *GLRX* may be specifically expressed in M0 macrophages compared to other types of macrophages. To verify this finding, IHC co-localization staining was performed to explore the expression of GLRX in macrophages in tumor specimens. A previous study reported that CD11b was a biomarker of M0 macrophages (17). The results showed that GLRX was expressed in most M0 macrophages (**Figures 7A, B**). Furthermore, GM-CSF and PMA were used to induce THP-1 cells to differentiate toward M0 macrophages *in vitro*. Under this circumstance, M-CSF was added to medium to induce M0 macrophages to polarize to M2 macrophages (16, 17). IF staining results showed that GLRX was highly expressed by M0 macrophages compared to M2 macrophages (**Figures 7C, D**). These findings suggest that the effect of *GLRX* on the immune system is mediated by M0 macrophages, further validating that *GLRX* plays a pivotal role in the immune response.

### *GLRX* Predicts Survival Outcome in Gliomas

**Figure 8:**
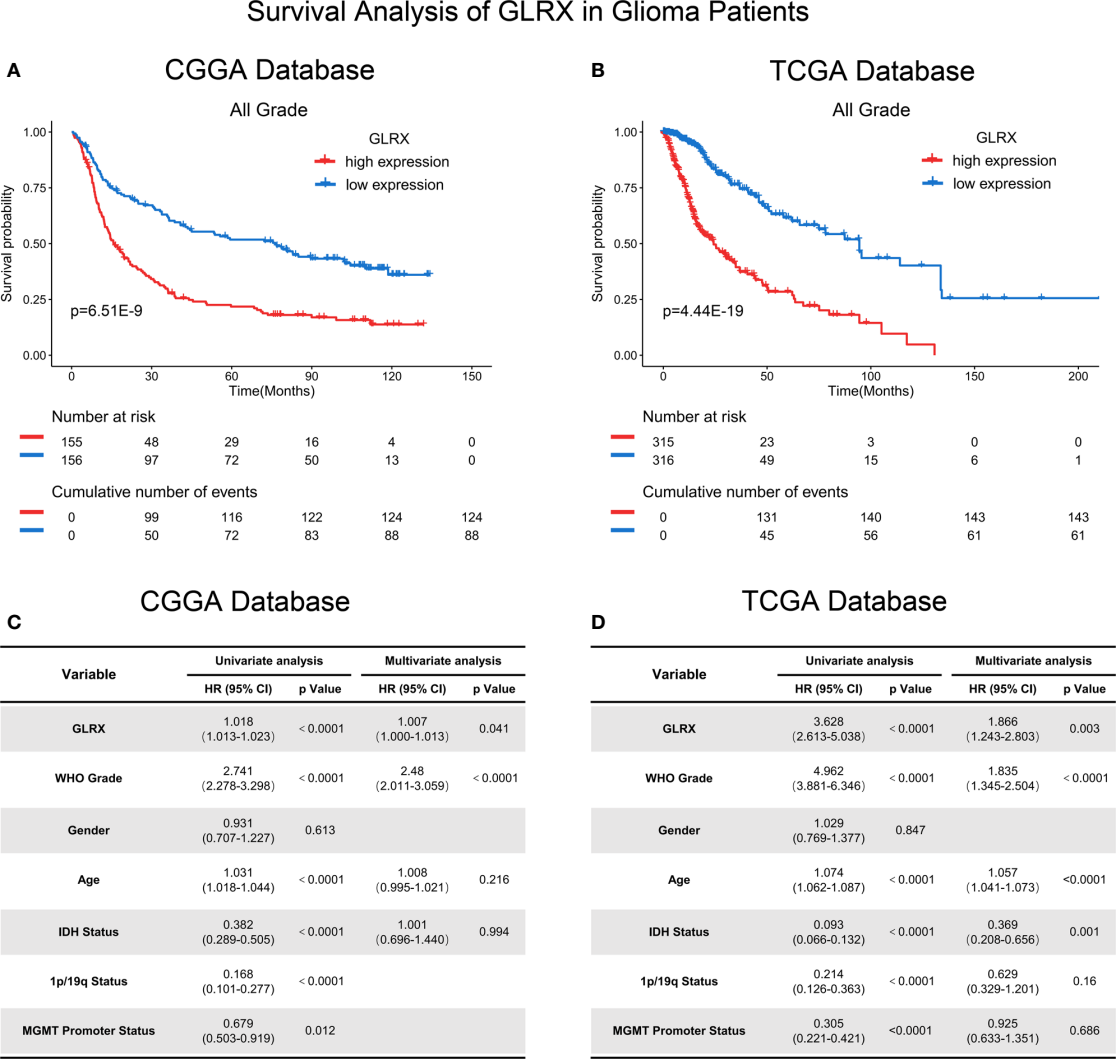
*GLRX* is a prognostic factor in glioma patients. **(A, B)** Clinical outcomes of patients with gliomas of low or high expression of *GLRX*. Kaplan-Meier survival analysis was performed in both CGGA and TCGA databases. **(C, D)** Univariate and multivariate analyses of clinical prognostic parameters in both CGGA and TCGA databases.

Because *GLRX* showed a robust negative correlation with the T cell response, we further investigated the prognostic value of *GLRX* by Kaplan-Meier and Cox proportional hazard model analyses. We found that patients with a higher expression of *GLRX* had a significantly shorter overall survival compared with those with lower *GLRX* expression (**Figures 8A, B** and **Supplementary Figure S7A**). Moreover, *GLRX* expression, WHO grade, age at diagnosis, *IDH* status, 1p/19q status, and MGMT promoter status were significantly associated with overall patient survival in all the three data sets that were evaluated. Multivariate analysis further confirmed *GLRX* as a significant predictor after adjusting for the clinical factors mentioned above (**Figures 8C, D** and **Supplementary Figure S7B**). These findings reveal that *GLRX* may serve as an indicator for the poor prognosis in gliomas due to its suppressive effects on the T cell immune response against tumor cells.

## Discussion

Glioma is one of the most fatal malignancies to afflict human health (1). Although temozolomide was approved for the treatment of gliomas by the U.S. Food and Drug Administration in 2005, researchers have continued to search for novel chemotherapeutic drugs with improved efficacy to treat gliomas (26). Nevertheless, no significant results have been achieved so far. Therefore, novel therapeutic approaches against gliomas remain an urgent requirement. In recent years, targeted drugs and immunotherapeutic approaches have exhibited extraordinary prospects (27, 28). Based on high-throughput sequencing, our team built the CGGA database and screened the *PTPRZ1*-*MET* fusion gene, which is expressed almost exclusively in secondary glioblastomas. The targeted drug PLB-1001 showed a good response rate in phase 2 clinical trials (29). Moreover, immune checkpoint blockade therapy also achieved success in treating gliomas. Cloughesy et al. reported that neoadjuvant anti-PD-1 immunotherapy, which enhances T cell–mediated antitumor immunity, could significantly extend overall survival of patients with recurrent glioblastomas (30). Despite the promising clinical results, these therapeutic approaches can benefit just a fraction of patients with gliomas. Therefore, exploring therapeutic approaches or a multifunctional small molecule that could benefit most glioma patients is of great interest.

The thioredoxin and glutathione systems are the key cellular redox systems involved in gliomas (8, 31, 32). Glioma proliferation is associated with parenchymal alterations and oxidative stress that further leads to the impairment of brain homeostasis (4). Tumor cellular respiration produces hyperoxides, such as H_2_O_2_, and reactive oxygen species (ROS), which, when present at high levels, damage the DNA. This process is considered to be a pernicious factor in malignant glioma development (33). Hence, functional antioxidant systems that can scavenge these hyperoxides hold promise to keep the cell cycle of glioma cells under control. Simultaneously, a better understanding of the antioxidant system can pave the way for finding new therapeutic approaches to fight gliomas. Because glioma cells are more susceptible to oxidative stress induced by hyperoxides, inhibition of antioxidant systems or their components can prevent them from performing oxidative scavenging, thereby exposing the glioma cells to intense oxidative stress and blocking their proliferation, leading to their death. The endogenous antioxidative molecule Grx plays an important role in the glutathione system (7, 34). Grx expression is associated with tumor proliferation and therapy resistance in several cancers. Previous studies report that Grx is overexpressed in pancreatic ductal carcinoma compared to normal pancreatic tissue and that Grx overexpression increases MCF-7 adenocarcinoma cell resistance to doxorubicin (35, 36). However, little is known about the role of Grx in gliomas. Therefore, as a potential therapeutic target, it is imperative to explore the unique role of Grx and how it works in gliomas.

We started by checking the expression of *GLRX* in glioblastoma tissue compared with normal brain tissue. Our results showed that, similar to other tumor types, glioblastoma samples exhibited higher expression of *GLRX*. Next, we analyzed RNA-seq data of 1,717 glioma patients compiled from the CGGA, CGGA (2019), and TCGA databases. As expected, *GLRX* expression was significantly upregulated in higher malignant pathological grades of gliomas. Moreover, we also found that *GLRX* expression was significantly higher in glioma patients with malignant molecular phenotypes, including those harboring the *IDH* wild-type state, 1p/19q non-codeletion state, and MGMT unmethylated promoters. Furthermore, *GLRX* was highly enriched in mesenchymal subtype gliomas. The mesenchymal subtype is characterized by stronger immunosuppression, and aggressive phenotype, and malignant proliferation due to the mesenchymal differentiation triggered by *NF1* mutations (21). A previous study has reported enhanced expression of immune checkpoints in mesenchymal subtype cancers compared with the other three transcriptional characteristic subtypes (37). Therefore, *GLRX* may be upregulated and involved in the immunosuppressive microenvironment of gliomas *via* modulation of the cellular component of the immune system. These findings suggest that *GLRX* expression is associated with the malignant behavior of gliomas. Thus, unraveling the mechanism of *GLRX* in gliomas may pave the way for the development of novel therapeutic approaches to fight this deadly malignancy.

To gain an in-depth understanding of the biological functions of *GLRX*, a series of analyses were performed. GO analysis revealed that *GLRX* plays a crucial role in immune and inflammatory responses in gliomas. Consistent with these results, KEGG and GSVA analyses also show that *GLRX* and related genes are involved in several immune response pathways, and *GLRX* is positively correlated with most immune functions with the exception of T cell response. Furthermore, *GLRX* was found to be significantly enriched in the mesenchymal glioma subtype with *GLRX* negatively mediating the T cell response and playing a suppressive role in the antitumor immune response. Taken together, these results suggest that *GLRX* may upregulate the expression of immune checkpoints to perform these functions. Upon analysis of the relationship between *GLRX* and known immune checkpoint genes, we confirm that *GLRX* is positively correlated with most inhibitory immune checkpoints, including PD-1, TIM-3, PD-L1, and PD-L2, which are involved in the regulation of the PD-1/PD-L1 pathway. These immune checkpoints are major negative immune regulators and are involved in regulating T cell activation, tolerance, and exhaustion (19). Our findings demonstrate that *GLRX* may exert antiglioma immune roles by affecting the expression of these inhibitory immune checkpoints. Additionally, *GLRX* is involved in inflammatory activities known to promote glioma progression *via* activation of tumor-associated macrophages (14, 38). To further validate the role of *GLRX* in the immune response, we used CIBERSORT software to calculate the percentage of each type of infiltrated immune cell. Our results show that *GLRX* is positively correlated with macrophages but negatively correlated with different subgroups of T cells. This further confirms the conclusions of our study. Last, single-cell sequencing analysis and IHC co-localization staining were performed to identify the exact components of the immune system that express *GLRX*. RNA-seq data and cellular molecular biomarkers reveal that *GLRX* is enriched in immune cells, particularly in M0 macrophages. M0 macrophages are a subgroup of resting immune cells that can undergo a directional polarization (17) to classically activated M1 macrophages and alternatively activated M2 macrophages. Macrophages in glioma tissue are prone to M2-like phenotypes, which are considered to be tumor-supporting macrophages (39). A previous study also reported that patients with higher expression of M0 macrophages had a poorer prognosis (17). Thus, we suspected that *GLRX* may play a role in M0 polarization and have an immuno-suppressive function. Based on the results of our present study, we hypothesize that *GLRX* is a potential target for redox and immunotherapy of gliomas.

Importantly, high levels of *GLRX* were associated with poor patient prognosis. Univariate and multivariate analyses indicated that high expression of *GLRX* predicted significantly lower survival. As a result, *GLRX* may serve as a potential prognostic predictor for glioma patients.

Redox therapy is being increasingly explored in tumor therapy (40, 41). Studies on breast, liver, pancreatic, and non-small cell lung cancers report that blocking the glutathione system could prevent tumor cell proliferation *in vitro* and *in vivo* (33, 35, 36, 42). As gliomas have access to abundant oxygen as well as to cellular respiration products, glioma cells become more dependent on the antioxidant system to survive and proliferate. Even a slight reduction in antioxidant levels could lead to glioma cell death (43). Meanwhile, cancer immunotherapy has also shown potential benefits for glioma patients. CAR-T, anti-PD-1, and anti-PD-L1 immunotherapies have shown higher immune response rates and longer survival in patients with brain metastases (2, 24, 27, 30). Our study suggests that *GLRX* is a key regulator of immune checkpoints and the immune response. Therefore, as a co-regulator of both redox and immune systems, inhibiting Grx could not only kill glioma cells through directly enhancing oxidative stress, but also downregulate the expression of inhibitory immune checkpoints and enhance the immune response. Thus, our study establishes *GLRX* as a novel potential target to enhance the efficacy of anticancer therapies, thereby paving the way for novel therapeutic approaches for treating gliomas.

## Data Availability Statement

The datasets presented in this study can be found in online repositories. The names of the repository/repositories and accession number(s) can be found at: https://www.cgga.org.cn, CGGA.

## Ethics Statement

The studies involving human participants were reviewed and approved by Capital Medical University Institutional Review Board (IRB). The patients/participants provided their written informed consent to participate in this study.

## Author Contributions

YC, GL: data analysis and editing the manuscript. YZ, LH: data collection and organization of CGGA database. YF, DW: data collection and organization of TCGA database. WZ, HH: conception, supervision, and design of the manuscript. All authors contributed to the article and approved the submitted version.

## Funding

This work was supported by grants from the National Natural Science Foundation of China (No. 81972816, 81672479).

## Conflict of Interest

The authors declare that the research was conducted in the absence of any commercial or financial relationships that could be construed as a potential conflict of interest.

## Supplementary Material

Supplementary Figure 1 GLRX is correlated with the relative malignant molecular pathological characteristics of glioma. **(A)** GLRX was significantly increased in GBM (WHO grade IV) in the CGGA (2019) database. **(B)** GLRX was significantly increased in IDH wild-type gliomas in the CGGA (2019) database (Mut: IDH mutation; WT: IDH wild type). **(C)** GLRX was significantly increased in 1p/19q non-co-deletion gliomas in the CGGA (2019) database (Codel: 1p/19q co-deletion; Non-codel: 1p/19q non-co-deletion). **(D)** GLRX was significantly increased in the MGMT unmethylated group in the CGGA (2019) database. ns and **** indicate no statistical difference and p < 0.0001, respectively.

Supplementary Figure 2 GLRX is a potential marker for malignant subtypes of gliomas. **(A)** GLRX was highly expressed in the mesenchymal subtype in the CGGA (2019) database. **(B)** ROC curve analysis showed that GLRX was highly sensitive and specific to predict the mesenchymal subtype in the CGGA (2019) database. **(C–E)** ROC curve analysis showed that BMI1 was less sensitive and specific to predict the mesenchymal subtype in the CGGA, TCGA, and CGGA (2019) databases, respectively. **(F–H)** ROC curve analysis showed that CD44 was highly sensitive and specific to predict the mesenchymal subtype in the CGGA, TCGA, and CGGA (2019) databases, respectively. Differences between groups were tested by Tukey’s multiple comparisons test. *** and **** indicate p < 0.001 and p < 0.0001, respectively.

Supplementary Figure 3 GLRX is strongly associated with immune processes in gliomas. **(A)** GO analysis showed that GLRX was mostly associated with immune, defense, and inflammatory responses in the CGGA (2019) database. **(B)** KEGG pathway analysis showed that GLRX was mostly involved in the immune response–related pathway in the CGGA (2019) database. **(C)** Most immune process–related genes were significantly positively correlated with GLRX expression in the CGGA (2019) database.

Supplementary Figure 4 GLRX is closely related to the state of tumor immune functions. **(A, B)** GLRX had positive correlation with 87.07% of the biological functions of the immune system process in the CGGA (2019) database. The scale values in the graph represent the proportions of significantly correlated biological functions in each biological function classification. **(B)** The correlation coefficient between GLRX and the immune function scores in the CGGA (2019) database. The red words represent a positive correlation. The green words represent a negative correlation.

Supplementary Figure 5 GLRX is associated with immune checkpoints and inflammatory activities. **(A–D)** GLRX was synergistic with inhibitory immune checkpoints in tumor-induced immune responses. A strong correlation between GLRX and inhibitory immune checkpoint expression was found in the CGGA (2019) database. **(E)** The correlation coefficient between GLRX and inflammatory activity function scores in the CGGA (2019) database. The red circle represents a positive correlation. The blue circle represents a negative correlation. The grey “×” represents no significant correlation.

Supplementary Figure 6 The relationship between GLRX and infiltrated immune cells in the CGGA (2019) database. ns, *, **, ***, and **** indicate no statistical difference, p < 0.05, p < 0.01, p < 0.001, and p<0.0001, respectively.

Supplementary Figure 7 GLRX is a prognostic factor in glioma patients. **(A)** Clinical outcomes of patients with gliomas of low or high expression of GLRX. Kaplan-Meier survival analysis was performed in the CGGA database. **(B)** Univariate and multivariate analyses of clinical prognostic parameters in CGGA database.

## References

[1] Jiang T Mao Y Ma W Mao Q You Y Yang X CGCG clinical practice guidelines for the management of adult diffuse gliomas. Cancer Lett (2016) 375(2):263–73. 10.1016/j.canlet.2016.01.024 26966000

[2] Van Meir EG Hadjipanayis CG Norden AD Shu HK Wen PY Olson JJ Exciting new advances in neuro-oncology: the avenue to a cure for malignant glioma. CA Cancer J Clin (2010) 60(3):166–93. 10.3322/caac.20069 PMC2888474 20445000

[3] Ahn BY Moss B Glutaredoxin Homolog Encoded by Vaccinia Virus Is a Virion-Associated Enzyme with Thioltransferase and Dehydroascorbate Reductase Activities. Proc Natl Acad Sci U S A (1992) 89(15):7060–4. 10.1073/pnas.89.15.7060 PMC49645 1496000

[4] Branco V Pimentel J Brito MA Carvalho C Thioredoxin, Glutathione and Related Molecules in Tumors of the Nervous System. Curr Med Chem (2020) 27(12):1878–900. 10.2174/0929867326666190201113004 30706774

[5] Du YT Zhang HH Lu J Holmgren A Glutathione and Glutaredoxin Act as a Backup of Human Thioredoxin Reductase 1 to Reduce Thioredoxin 1 Preventing Cell Death by Aurothioglucose. J Biol Chem (2012) 287(45):38210–9. 10.1074/jbc.M112.392225 PMC3488090 22977247

[6] Backos DS Franklin CC Reigan P The role of glutathione in brain tumor drug resistance. Biochem Pharmacol (2012) 83(8):1005–12. 10.1016/j.bcp.2011.11.016 22138445

[7] Ren XY Zou LL Zhang X Branco V Wang J Carvalho C Redox Signaling Mediated by Thioredoxin and Glutathione Systems in the Central Nervous System. Antioxid Redox Signaling (2017) 27(13):989–1010. 10.1089/ars.2016.6925 PMC5649126 28443683

[8] Zhang PX Gao JX Wang X Wen WH Yang HW Tian YJ A novel indication of thioredoxin-interacting protein as a tumor suppressor gene in malignant glioma. Oncol Lett (2017) 14(2):2053–8. 10.3892/ol.2017.6397 PMC5530178 28781647

[9] Chai RC Zhang KN Chang YZ Wu F Liu YQ Zhao Z Systematically characterize the clinical and biological significances of 1p19q genes in 1p/19q non-codeletion glioma. Carcinogenesis (2019) 40(10):1229–39. 10.1093/carcin/bgz102 31157866

[10] Chai RC Zhang KN Liu YQ Wu F Zhao Z Wang KY Combinations of four or more CpGs methylation present equivalent predictive value for MGMT expression and temozolomide therapeutic prognosis in gliomas. CNS Neurosci Ther (2019) 25(3):314–22. 10.1111/cns.13040 PMC6488893 30117294

[11] Wang HY Tang K Liang TY Zhang WZ Li JY Wang W The comparison of clinical and biological characteristics between IDH1 and IDH2 mutations in gliomas. J Exp Clin Cancer Res (2016) 35:86. 10.1186/s13046-016-0362-7 27245697 PMC4888668

[12] Tang ZF Li CW Kang BX Gao G Li C Zhang ZM GEPIA: a web server for cancer and normal gene expression profiling and interactive analyses. Nucleic Acids Res (2017) 45(W1):W98–W102. 10.1093/nar/gkx247 28407145 PMC5570223

[13] Huang DW Sherman BT Lempicki RA Systematic and integrative analysis of large gene lists using DAVID bioinformatics resources. Nat Protoc (2009) 4(1):44–57. 10.1038/nprot.2008.211 19131956

[14] Li GZ Wang Z Zhang CB Liu X Cai JQ Wang ZL Molecular and clinical characterization of TIM-3 in glioma through 1,024 samples. Oncoimmunology (2017) 6(8):e1328339. 10.1080/2162402X.2017.1328339 PMC5593703 28919992

[15] Newman AM Steen CB Liu CL Gentles AJ Chaudhuri AA Scherer F Determining cell type abundance and expression from bulk tissues with digital cytometry. Nat Biotechnol (2019) 37(7):773–+. 10.1038/s41587-019-0114-2 PMC6610714 31061481

[16] Knowles LM Kagiri D Bernard M Schwarz EC Eichler H Pilch J Macrophage Polarization is Deregulated in Haemophilia. Thromb Haemostasis (2019) 119(2):234–45. 10.1055/s-0038-1676796 30650445

[17] Gabrusiewicz K Rodriguez B Wei J Hashimoto Y Healy LM Maiti SN Glioblastoma-infiltrated innate immune cells resemble M0 macrophage phenotype. Jci Insight (2016) 1(2):e85841. 10.1172/jci.insight.85841 PMC4784261 26973881

[18] Wang Q He Z Huang M Liu T Wang Y Xu H Vascular niche IL-6 induces alternative macrophage activation in glioblastoma through HIF-2alpha. Nat Commun (2018) 9(1):559. 10.1038/s41467-018-03050-0 29422647 PMC5805734

[19] Wang Z Zhang CB Liu X Wang ZL Sun LH Li GZ Molecular and clinical characterization of PD-L1 expression at transcriptional level via 976 samples of brain glioma. OncoImmunology (2016) 5(11):e1196310. 10.1080/2162402X.2016.1196310 PMC5139638 27999734

[20] Hartmann C Meyer J Balss J Capper D Mueller W Christians A Type and frequency of IDH1 and IDH2 mutations are related to astrocytic and oligodendroglial differentiation and age: a study of 1,010 diffuse gliomas. Acta Neuropathol (2009) 118(4):469–74. 10.1007/s00401-009-0561-9 19554337

[21] Verhaak RGW Hoadley KA Purdom E Wang V Qi Y Wilkerson MD Integrated Genomic Analysis Identifies Clinically Relevant Subtypes of Glioblastoma Characterized by Abnormalities in PDGFRA, IDH1, EGFR, and NF1. Cancer Cell (2010) 17(1):98–110. 10.1016/j.ccr.2009.12.020 20129251 PMC2818769

[22] Behnan J Finocchiaro G Hanna G The landscape of the mesenchymal signature in brain tumours. Brain (2019) 142:847–66. 10.1093/brain/awz044 PMC6485274 30946477

[23] Jin X Kim L Wu Q Wallace LC Prager B Sanvoranart T Targeting Glioma Stem Cells through Combined Bmi1 and Ezh2 Inhibition. Neuro-Oncology (2017) 19:80–0. 10.1093/neuonc/nox168.329 PMC5679732 29035367

[24] Zhao XD Subramanian S Oncogenic pathways that affect antitumor immune response and immune checkpoint blockade therapy. Pharmacol Ther (2018) 181:76–84. 10.1016/j.pharmthera.2017.07.004 28720430

[25] Wang ZL Wang Z Li GZ Wang QW Bao ZS Zhang CB Immune Cytolytic Activity Is Associated With Genetic and Clinical Properties of Glioma. Front Immunol (2019) 10:1756. 10.3389/fimmu.2019.01756 31428092 PMC6688525

[26] Herrlinger U Tzaridis T Mack F Steinbach JP Schlegel U Sabel M Lomustine-temozolomide combination therapy versus standard temozolomide therapy in patients with newly diagnosed glioblastoma with methylated MGMT promoter (CeTeG/NOA-09): a randomised, open-label, phase 3 trial. Lancet (2019) 393(10172):678–88. 10.1016/S0140-6736(18)31791-4 30782343

[27] Sharma P Allison JP The future of immune checkpoint therapy. Science (2015) 348(6230):56–61. 10.1126/science.aaa8172 25838373

[28] Zhu XF Zhou H Liu YN Wen YY Wei CF Yu QQ Transferrin/aptamer conjugated mesoporous ruthenium nanosystem for redox-controlled and targeted chemo-photodynamic therapy of glioma. Acta Biomater (2018) 82:143–57. 10.1016/j.actbio.2018.10.012 30316026

[29] Hu H Mu Q Bao Z Chen Y Liu Y Chen J Mutational Landscape of Secondary Glioblastoma Guides MET-Targeted Trial in Brain Tumor. Cell (2018) 175(6):1665–1678 e1618. 10.1016/j.cell.2018.09.038 30343896

[30] Cloughesy TF Mochizuki AY Orpilla JR Hugo W Lee AH Davidson TB Neoadjuvant anti-PD-1 immunotherapy promotes a survival benefit with intratumoral and systemic immune responses in recurrent glioblastoma. Nat Med (2019) 25(3):477–+. 10.1038/s41591-018-0337-7 PMC6408961 30742122

[31] Branco V Coppo L Sola S Lu J Rodrigues CMP Holmgren A Impaired cross-talk between the thioredoxin and glutathione systems is related to ASK-1 mediated apoptosis in neuronal cells exposed to mercury. Redox Biol (2017) 13:278–87. 10.1016/j.redox.2017.05.024 PMC5466585 28600984

[32] Yokomizo A Ono M Nanri H Makino Y Ohga T Wada M Cellular levels of thioredoxin associated with drug sensitivity to cisplatin, mitomycin C, doxorubicin, and etoposide. Cancer Res (1995) 55(19):4293–6. 7671238

[33] Zhang QQ Chen WQ Lv XL Weng QY Chen MJ Cui R Piperlongumine, a Novel TrxR1 Inhibitor, Induces Apoptosis in Hepatocellular Carcinoma Cells by ROS-Mediated ER Stress. Front Pharmacol (2019) 10:1180. 10.3389/fphar.2019.01180 31680962 PMC6802400

[34] Okuda M Inoue N Azumi H Seno T Sumi Y Hirata K Expression of glutaredoxin in human coronary arteries: its potential role in antioxidant protection against atherosclerosis. Arterioscler Thromb Vasc Biol (2001) 21(9):1483–7. 10.1161/hq0901.095550 11557676

[35] Meyer EB Wells WW Thioltransferase overexpression increases resistance of MCF-7 cells to adriamycin. Free Radical Biol Med (1999) 26(5-6):770–6. 10.1016/S0891-5849(98)00247-0 10218667

[36] Nakamura H Bai J Nishinaka Y Ueda S Sasada T Ohshio G Expression of thioredoxin and glutaredoxin, redox-regulating proteins, in pancreatic cancer. Cancer Detection Prev (2000) 24(1):53–60. 10757123

[37] Doucette T Rao G Rao A Shen L Aldape K Wei J Immune Heterogeneity of Glioblastoma Subtypes: Extrapolation from the Cancer Genome Atlas. Cancer Immunol Res (2013) 1(2):112–22. 10.1158/2326-6066.Cir-13-0028 PMC3881271 24409449

[38] Guan XD Zhang CB Zhao JY Sun G Song QK Jia W CMTM6 overexpression is associated with molecular and clinical characteristics of malignancy and predicts poor prognosis in gliomas. Ebiomedicine (2018) 35:233–43. 10.1016/j.ebiom.2018.08.012 PMC6156716 30131308

[39] Xiang W Shi RC Kang X Zhang X Chen P Zhang LL Monoacylglycerol lipase regulates cannabinoid receptor 2-dependent macrophage activation and cancer progression. Nat Commun (2018) 9(1):2574. 10.1038/s41467-018-04999-8 PMC6030061 29968710

[40] Salazar-Ramiro A Ramirez-Ortega D de la Cruz VP Hernandez-Pedro NY Gonzalez-Esquivel DF Sotelo J Role of Redox Status in Development of Glioblastoma. Front Immunol (2016) 7:156. 10.3389/fimmu.2016.00156 27199982 PMC4844613

[41] Trachootham D Alexandre J Huang P Targeting cancer cells by ROS-mediated mechanisms: a radical therapeutic approach? Nat Rev Drug Discovery (2009) 8(7):579–91. 10.1038/nrd2803 19478820

[42] Peltoniemi MJ Rytila PH Harju TH Soini YM Salmenkivi KM Ruddock LW Modulation of glutaredoxin in the lung and sputum of cigarette smokers and chronic obstructive pulmonary disease. Respir Res (2006) 7:133. 10.1186/1465-9921-7-133 17064412 PMC1633737

[43] Durgadoss L Nidadavolu P Valli RK Saeed U Mishra M Seth P Redox modification of Akt mediated by the dopaminergic neurotoxin MPTP, in mouse midbrain, leads to down-regulation of pAkt. FASEB J (2012) 26(4):1473–83. 10.1096/fj.11-194100 22198382

