# Supplementary figures and images for: Redox Regulator GLRX Is Associated With Tumor Immunity in Glioma
Source: Front Immunol. 2020 Nov 30;11:580934. doi: 10.3389/fimmu.2020.580934 (PMC7734322; doi:10.3389/fimmu.2020.580934)

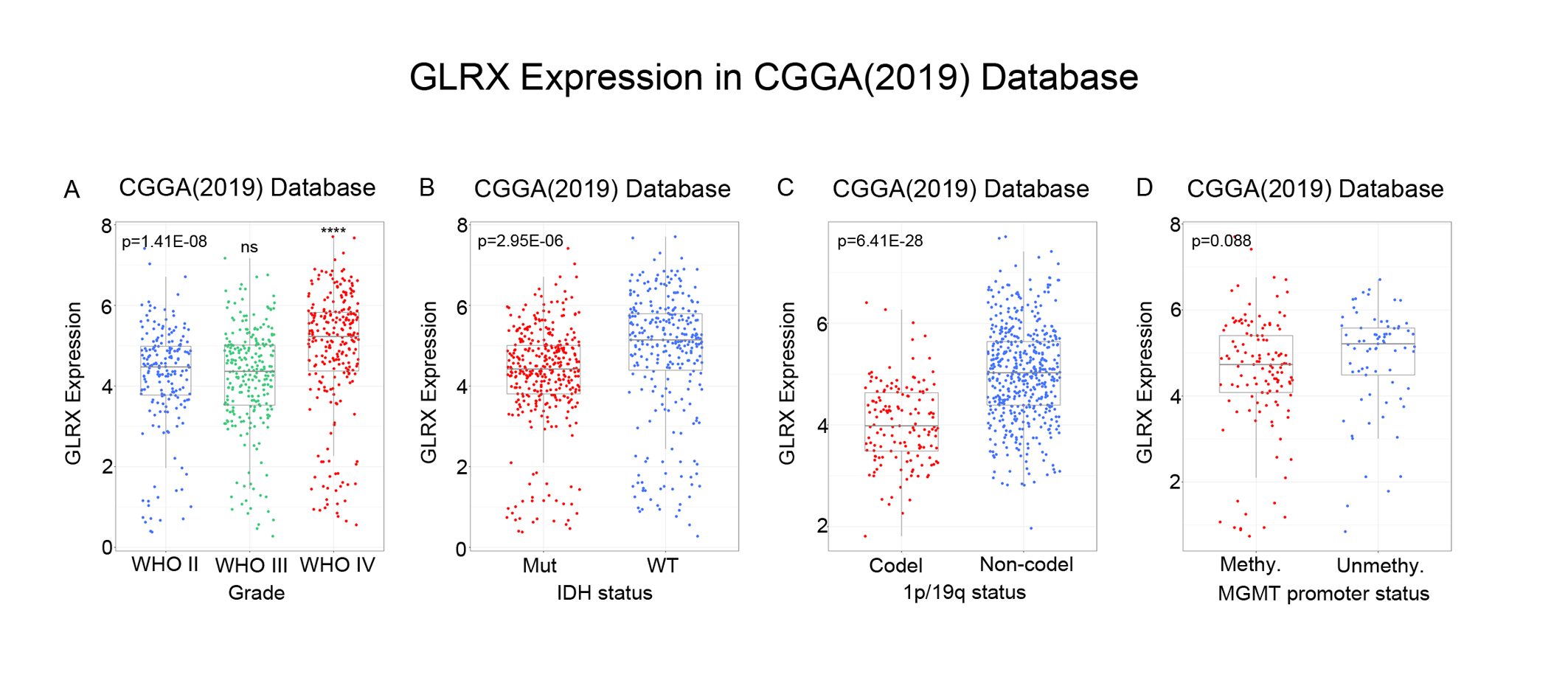

Supplement: Supplementary Figure 1 — GLRX is correlated with the relative malignant molecular pathological characteristics of glioma. (A) GLRX was significantly increased in GBM (WHO grade IV) in the CGGA (2019) database. (B) GLRX was significantly increased in IDH wild-type gliomas in the CGGA (2019) database (Mut: IDH mutation; WT: IDH wild type). (C) GLRX was significantly increased in 1p/19q non-co-deletion gliomas in the CGGA (2019) database (Codel: 1p/19q co-deletion; Non-codel: 1p/19q non-co-deletion). (D) GLRX was significantly increased in the MGMT unmethylated group in the CGGA (2019) database. ns and **** indicate no statistical difference and p < 0.0001, respectively. [file Image_1.tif]

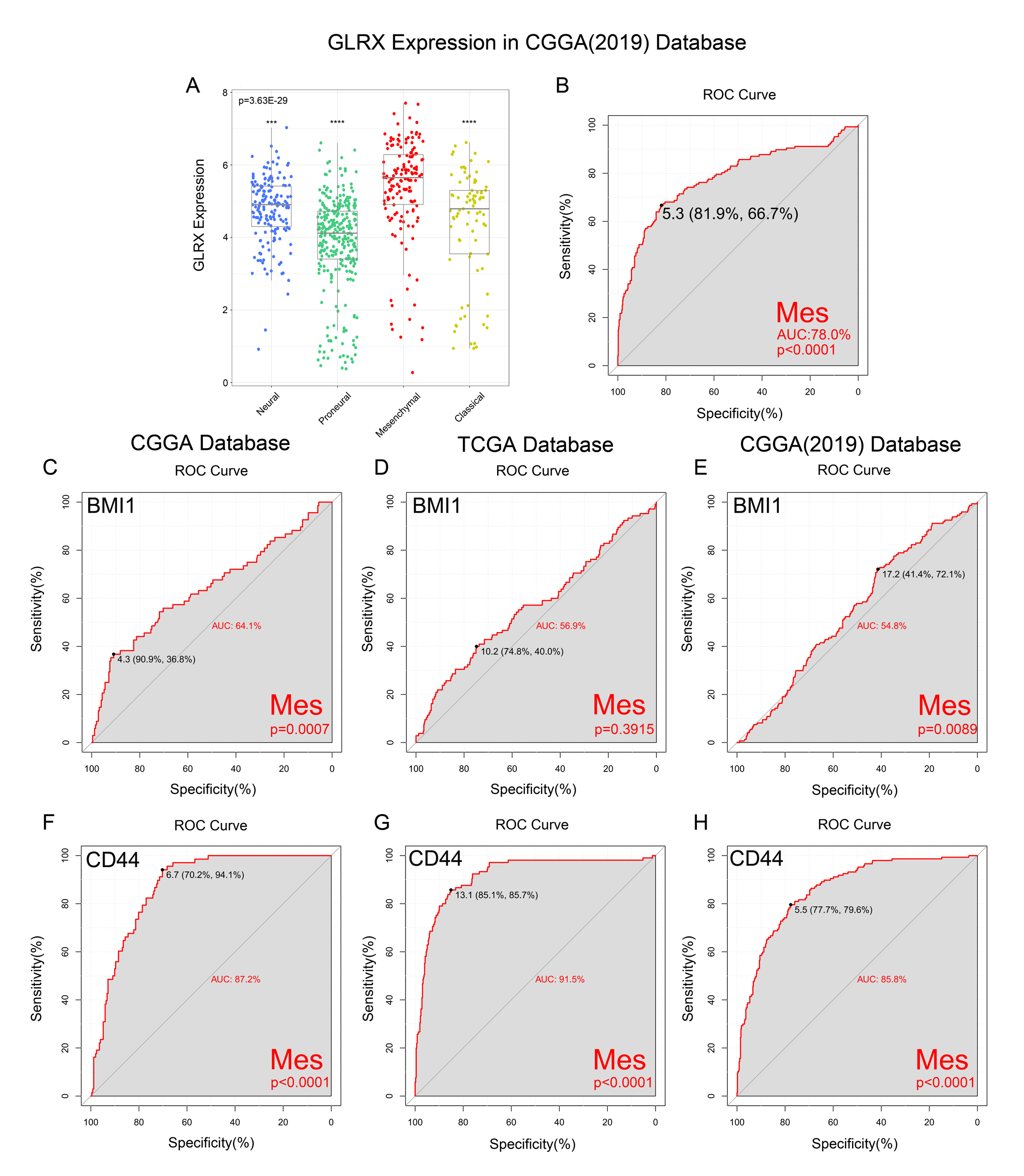

Supplement: Supplementary Figure 2 — GLRX is a potential marker for malignant subtypes of gliomas. (A) GLRX was highly expressed in the mesenchymal subtype in the CGGA (2019) database. (B) ROC curve analysis showed that GLRX was highly sensitive and specific to predict the mesenchymal subtype in the CGGA (2019) database. (C–E) ROC curve analysis showed that BMI1 was less sensitive and specific to predict the mesenchymal subtype in the CGGA, TCGA, and CGGA (2019) databases, respectively. (F–H) ROC curve analysis showed that CD44 was highly sensitive and specific to predict the mesenchymal subtype in the CGGA, TCGA, and CGGA (2019) databases, respectively. Differences between groups were tested by Tukey’s multiple comparisons test. *** and **** indicate p < 0.001 and p < 0.0001, respectively. [file Image_2.tif]

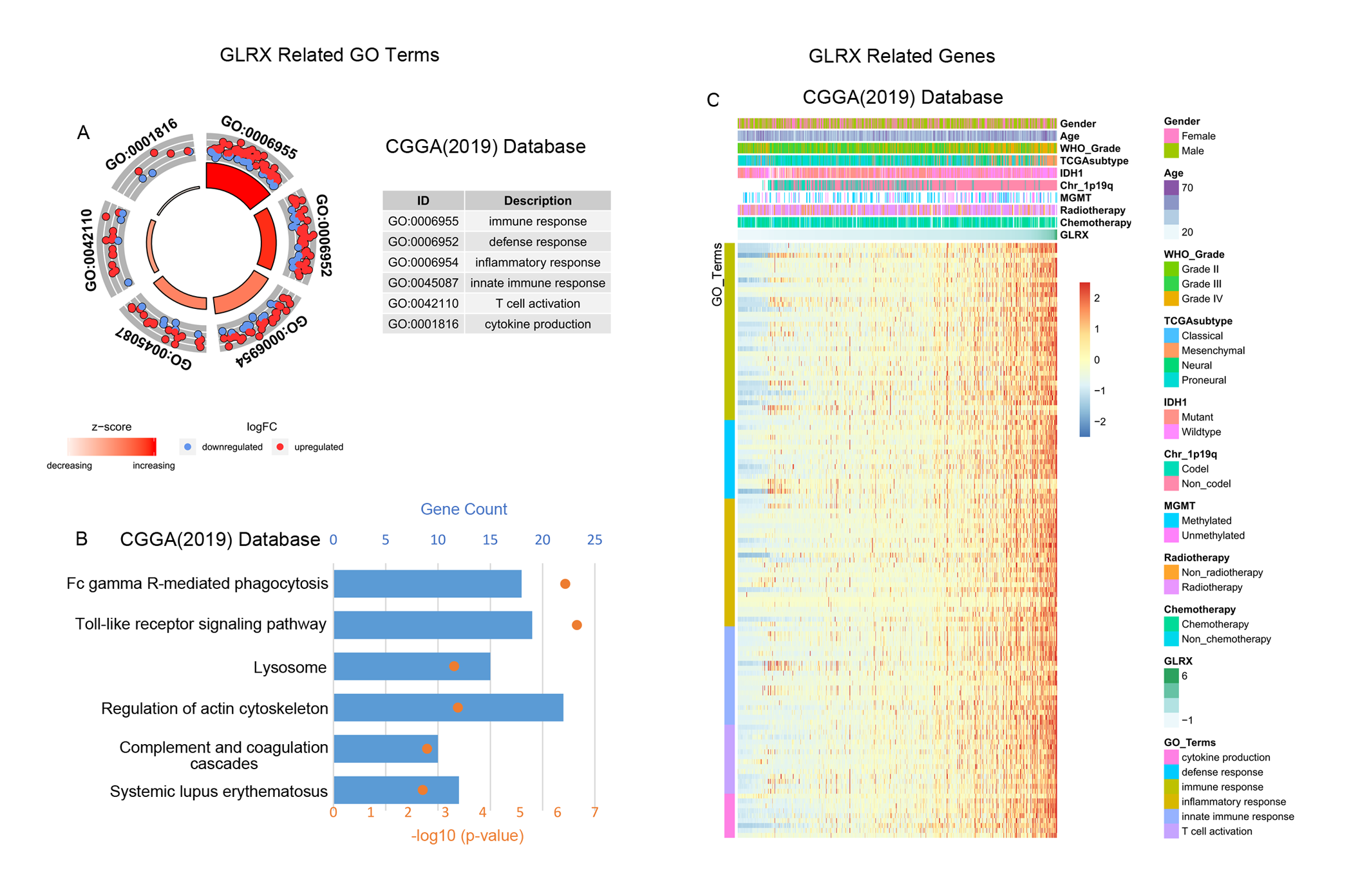

Supplement: Supplementary Figure 3 — GLRX is strongly associated with immune processes in gliomas. (A) GO analysis showed that GLRX was mostly associated with immune, defense, and inflammatory responses in the CGGA (2019) database. (B) KEGG pathway analysis showed that GLRX was mostly involved in the immune response–related pathway in the CGGA (2019) database. (C) Most immune process–related genes were significantly positively correlated with GLRX expression in the CGGA (2019) database. [file Image_3.tif]

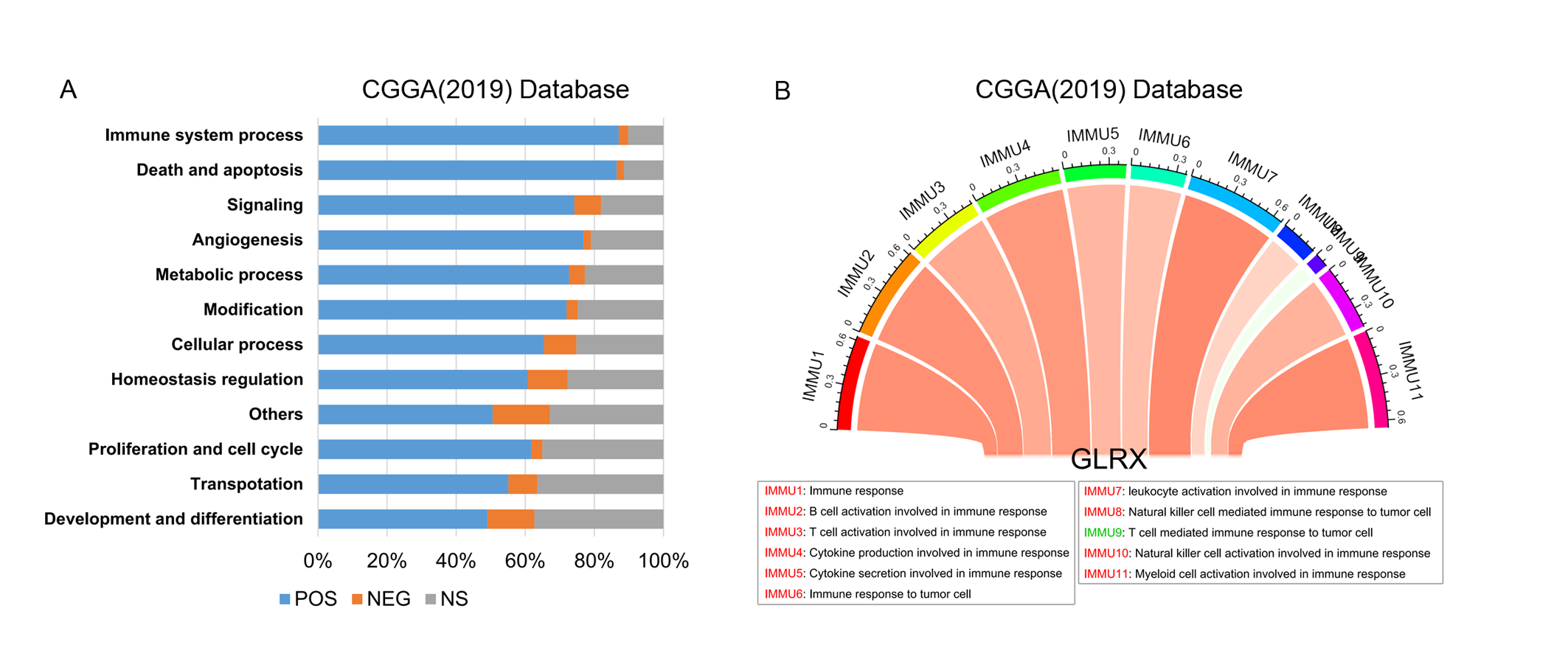

Supplement: Supplementary Figure 4 — GLRX is closely related to the state of tumor immune functions. (A, B) GLRX had positive correlation with 87.07% of the biological functions of the immune system process in the CGGA (2019) database. The scale values in the graph represent the proportions of significantly correlated biological functions in each biological function classification. (B) The correlation coefficient between GLRX and the immune function scores in the CGGA (2019) database. The red words represent a positive correlation. The green words represent a negative correlation. [file Image_4.tif]

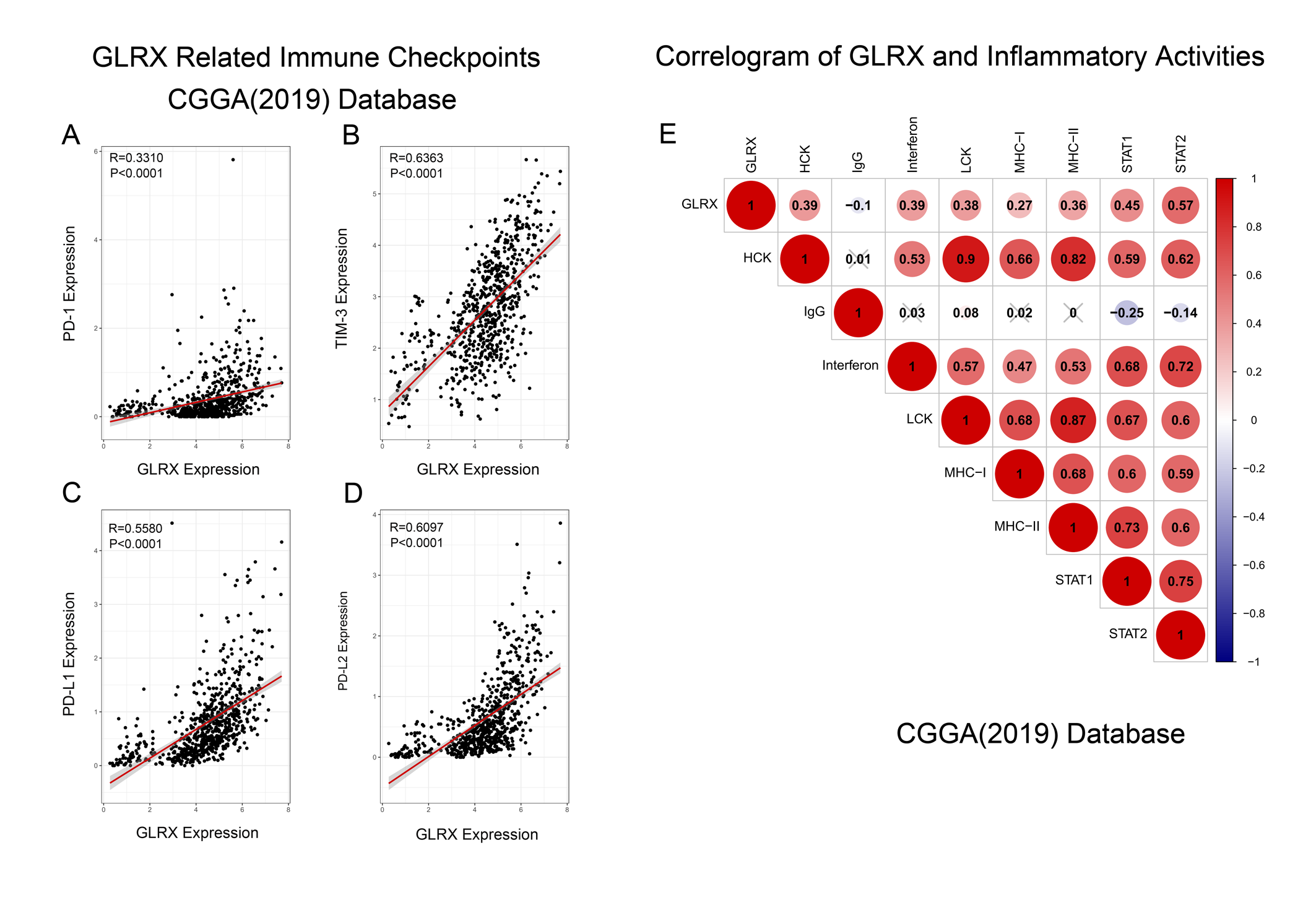

Supplement: Supplementary Figure 5 — GLRX is associated with immune checkpoints and inflammatory activities. (A–D) GLRX was synergistic with inhibitory immune checkpoints in tumor-induced immune responses. A strong correlation between GLRX and inhibitory immune checkpoint expression was found in the CGGA (2019) database. (E) The correlation coefficient between GLRX and inflammatory activity function scores in the CGGA (2019) database. The red circle represents a positive correlation. The blue circle represents a negative correlation. The grey “×” represents no significant correlation. [file Image_5.tif]

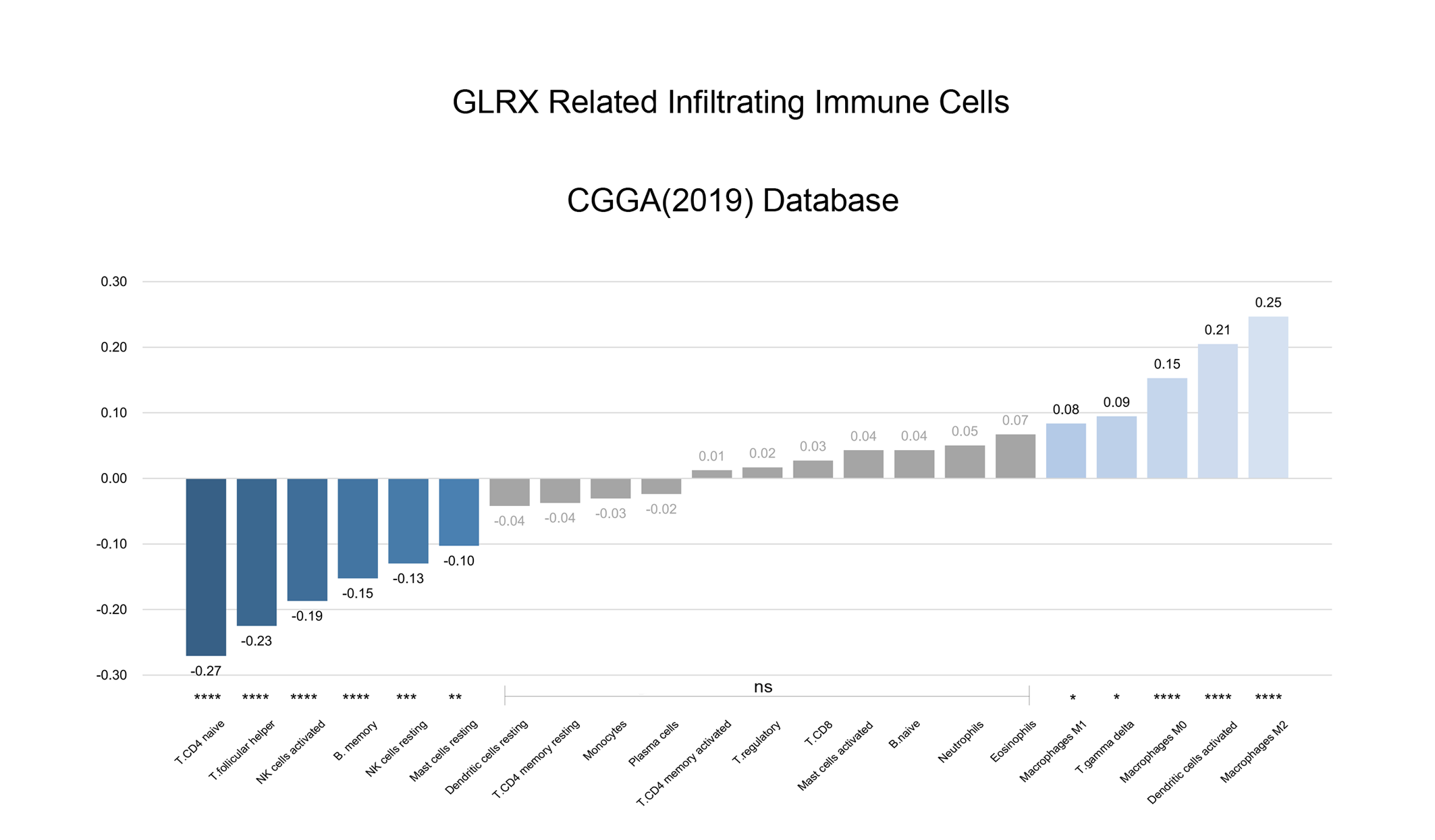

Supplement: Supplementary Figure 6 — The relationship between GLRX and infiltrated immune cells in the CGGA (2019) database. ns, *, **, ***, and **** indicate no statistical difference, p < 0.05, p < 0.01, p < 0.001, and p<0.0001, respectively. [file Image_6.tif]

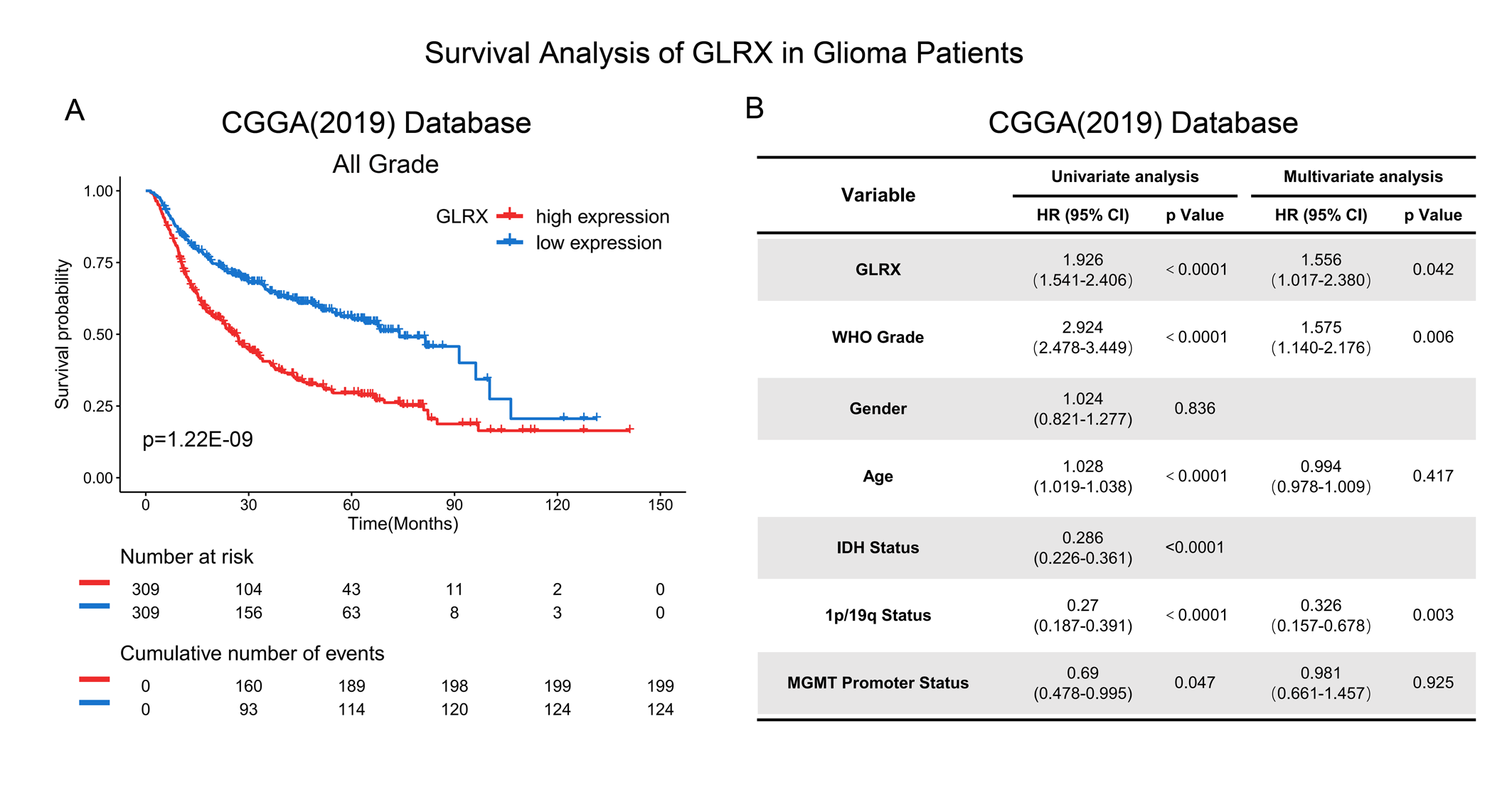

Supplement: Supplementary Figure 7 — GLRX is a prognostic factor in glioma patients. (A) Clinical outcomes of patients with gliomas of low or high expression of GLRX. Kaplan-Meier survival analysis was performed in the CGGA database. (B) Univariate and multivariate analyses of clinical prognostic parameters in CGGA database. [file Image_7.tif]
